# Inference of phenotype-relevant transcriptional regulatory networks elucidates cancer type-specific regulatory mechanisms in a pan-cancer study

**DOI:** 10.1038/s41540-021-00169-7

**Published:** 2021-02-08

**Authors:** Amin Emad, Saurabh Sinha

**Affiliations:** 1 Department of Electrical and Computer Engineering, McGill University, Montreal, QC Canada; 2 Carl R. Woese Institute for Genomic Biology, University of Illinois at Urbana-Champaign, Urbana, IL USA; 3 Department of Computer Science, University of Illinois at Urbana-Champaign, Urbana, IL USA; 4 Cancer Center at Illinois, University of Illinois at Urbana-Champaign, Urbana, IL USA

**Keywords:** Regulatory networks, Software, Cancer

## Abstract

Reconstruction of transcriptional regulatory networks (TRNs) is a powerful approach to unravel the gene expression programs involved in healthy and disease states of a cell. However, these networks are usually reconstructed independent of the phenotypic (or clinical) properties of the samples. Therefore, they may confound regulatory mechanisms that are specifically related to a phenotypic property with more general mechanisms underlying the full complement of the analyzed samples. In this study, we develop a method called InPheRNo to identify “phenotype-relevant” TRNs. This method is based on a probabilistic graphical model that models the simultaneous effects of multiple transcription factors (TFs) on their target genes and the statistical relationship between the target genes’ expression and the phenotype. Extensive comparison of InPheRNo with related approaches using primary tumor samples of 18 cancer types from The Cancer Genome Atlas reveals that InPheRNo can accurately reconstruct cancer type-relevant TRNs and identify cancer driver TFs. In addition, survival analysis reveals that the activity level of TFs with many target genes could distinguish patients with poor prognosis from those with better prognosis.

## Introduction

**Fig. 1:**
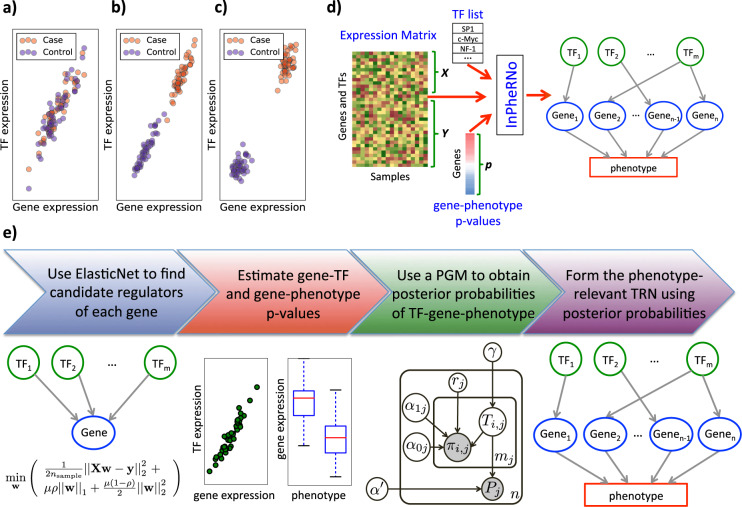
The phenotype-relevant TRN concept and an overview of the InPheRNo framework. **a** The scatter plot shows a scenario in which the gene–TF expression correlation (across different samples) is independent of the phenotype variation. **b**, **c** The scatter plots show two scenarios in which the gene–TF expression correlation is phenotype-relevant. **d** The inputs and outputs to InPheRNo are shown. The inputs include a matrix of gene expression for all genes (including TF genes), a list of TFs and a vector containing *p* value of gene–phenotype associations, denoted as ***p***. The list of TFs is used to divide the expression matrix into a matrix ***X*** of TF expressions and a matrix ***Y*** of gene expressions. As the output InPheRNo provides a phenotype-relevant TRN. **e** An overview of the InPheRNo pipeline is shown. First, the expression of genes and TFs are used in an Elastic Net algorithm to reduce the number of candidate TFs for each gene. Then, the pseudo *p* value of association between TF *i* and gene *j* (denoted by *π*_*i,j*_) is estimated using an OLS regression model that relates the expression of gene *j* to the expression of *m*_*j*_ candidate TFs. In addition, the *p* values of gene–phenotype associations (denoted by *Pj*) are assumed to be estimated and provided through ***p*** for *n* genes. These sets of *p* values are used as observed variables in a probabilistic graphical model to learn posterior probabilities for the (TF, gene, phenotype) triplets that a TF regulates a gene to affect the phenotype. These posterior probabilities are used to form the phenotype-relevant TRN.

Gene expression programs are responsible for many biological processes in a cell and extensive efforts have been devoted to elucidating these programs in healthy and disease states. Transcriptional regulatory networks (TRNs) have proven to be a useful framework for describing expression programs. A TRN is a network with transcription factors (TFs) and genes as nodes where a TF–gene edge represents a regulatory effect of the TF on the gene. TRNs are usually constructed from transcriptomic data across many conditions, alone or in combination with other data types^1,2^. Here, we are especially interested in methods for TRN reconstruction from expression data alone, owing to their broad applicability. The majority of such methods are agnostic of any phenotypic annotations of sampled conditions (e.g., case versus control status in disease studies, or drug sensitivity of cell lines in pharmacogenomics studies), looking only to capture correlations between TF and gene expression values in those conditions^3–7^. As a result, many edges in the reconstructed networks may not be particularly relevant to the phenotype being investigated by expression profiling. To take a simple example, consider the two scenarios of gene expression relationship between TF and gene shown in Fig. 1a and 1b. In both cases, a linear relationship is evident and is often interpreted as evidence for a TF–gene edge in the TRN. However, it is also apparent that the TF–gene relationship is potentially more related to the phenotypic class in the example of Fig. 1b than in the other example (Fig. 1a)—not only are the TF and gene expression levels correlated, but also the gene’s expression is clearly different between the classes in Fig. 1b, suggesting that the TF’s regulatory influence may underlie the expression variation between classes. A variant of the example of Fig. 1b is shown in Fig. 1c, which also illustrates a TF–gene relationship potentially related to the phenotypic distinction among samples. In this example, the expression levels of TF and gene are only weakly correlated in each phenotypic class (case or control) separately, possibly owing to noisy data and small sample sizes. Thus, methods that account for phenotypic class information by separately examining samples of each class may not detect this TF–gene relationship. However, when all the samples are considered simultaneously, the TF–gene relationship as well as its phenotype relevance becomes apparent. We believe there is a clear need for methods of TRN reconstruction that are geared towards detecting phenotype-relevant TF–gene relationships such as those idealized in Fig. 1b and 1c. Such methods will draw our attention to regulatory networks that control the variation of phenotypic scores/labels among different samples (e.g., case vs. control, subtypes of cancer, IC50 drug response values). In our definition of “phenotype-relevant TRNs”, a (TF, gene) edge implies evidence of regulation of the gene by the TF across all samples as well as evidence of association between the expression of the gene and the phenotypic label or score. We use the term phenotypic label/score to mean a priori known label or score assigned to each sample; for example, a phenotypic label could indicate whether the sample corresponds to case or control subject, and a phenotypic score could be the IC50 drug response value of a cell line.

Previous methods for including phenotypic information in TRN reconstruction fall under three major categories. The first approach is to restrict the analysis to samples of the same phenotypic label (e.g., a tissue type^8,9^ or a cancer type^10,11^). Although this approach, henceforth called “context-restricted” TRN reconstruction (Supplementary Fig. 1a in Supplementary Information) may identify important regulatory mechanisms relevant to a context, it does not solve the problem mentioned above—to reconstruct TRNs that may be responsible for the variation of the phenotypic values (or labels) of different samples. “Differential network analysis” or “DiNA” (Supplementary Fig. 1b in Supplementary Information) is another approach to relate TRNs to binary phenotypic labels. Here, two context-restricted networks are reconstructed based on samples from each of two phenotypic classes (e.g., case versus control), and a differential network is formed by comparing these two networks^12–16^. In focusing on the differential topology of regulatory networks, such methods may fail to identify important phenotype-relevant regulatory edges. For example, Fig. 1b and 1c illustrate TF–gene relationships that qualify as being “phenotype-relevant” by our definition above and perturbations that abolish them might affect the phenotype; however, such pairs are discarded by DiNA. The reason is that DiNA seeks TF–gene expression correlations that are present in one class exclusively (Supplementary Fig. 1b), whereas in our definition of phenotype-relevant relationships these correlations span all samples and it is the gene’s expression that differs between phenotypic classes. In addition, DiNA methods cannot be used with continuous-valued phenotypes, and become cumbersome even for categorical phenotypes with more than two categories. A third class of methods is that of “context-specific” network analysis (Supplementary Fig. 1c in Supplementary Information), in which genes associated with phenotype variation are identified, e.g., by differential expression analysis, and then a network is constructed by relating the expression of these genes to the expression of TFs^17–19^. In principle, such methods have the ability to detect the phenotype-relevant TF–gene relationships of Fig. 1b and 1c. However, one major disadvantage of this approach is that the phenotype relevance of genes is simply used as a filtering criterion based on arbitrary thresholds and its strength is ignored in TRN reconstruction. Our approach, in contrast, seeks to incorporate the extent of phenotype relevance of a gene, e.g., its differential expression between samples of different classes or its expression correlation with phenotypic scores, directly into the strength of TF–gene edges in the TRN. Finally, we note that methods that directly identify genes or TFs associated with a phenotype (including “master regulator analysis” or “MRA”)^20–23^ serve a different purpose and are not the focus of this study as they do not directly address the problem of reconstructing phenotype-relevant TRNs. In summary, TRNs are a highly useful and widely popular construct for characterizing gene expression programs underlying phenotypes, yet there is an urgent need for methods that incorporate phenotypic information directly into TRN reconstruction.

We report here a new computational method called InPheRNo (Inference of Phenotype-relevant Regulatory Networks) to reconstruct TRNs that help explain the variation in the phenotypic labels/scores of samples. It models the simultaneous effect of multiple TFs on their targets, as well as the association of target genes’ expression with samples’ phenotypic labels/scores. Its rigorous PGM can be used with categorical labels or continuous-valued phenotypic scores, and also provides a confidence score for the identified TF–gene regulatory edges. We applied InPheRNo to data from The Cancer Genome Atlas (TCGA)^24^ pertaining to 18 different cancer types, to reconstruct TRNs that differentiate one cancer type from other types of cancer. We also compared these TRNs to tissue-specific TRNs reconstructed by analysis of expression data from the Genotype-Tissue Expression Project (GTEx) project^25^, in order to make the former more specific to the cancer type. The resulting cancer type-relevant TRNs identified regulatory mechanisms involved in the development and progress of each cancer type and discerned previously known as well as novel cancer driver TFs that could be used as potential drug targets. In addition, survival analysis revealed that a gene expression signature formed using these TFs and their target genes can accurately distinguish between patients with poor prognosis and those with good prognosis for the majority of the cancer types. Finally, we applied InPheRNo to identify PAM50^26^ subtype-relevant TRNs in breast cancer patients (data obtained from TCGA) to show-case the application of this framework to non-binary phenotypic labels. We demonstrated the improved accuracy of InPheRNo-derived networks by comparing them to several baseline methods with respect to driver TF discovery and survival prediction. As transcriptomic profiling becomes a standard tool in the study of phenotypic variation among individuals^27^, the new tool presented here will help distill the associated high-dimensional information into specific regulatory mechanisms underlying that variation.

## Results

### A new probabilistic method for phenotype-relevant TRN reconstruction

We developed a computational method called InPheRNo to reconstruct phenotype-relevant TRNs by analyzing gene expression profiles of a set of samples along with associated phenotypic scores or labels of those samples. As noted in Introduction (also Fig. 1a–c), the key idea is to combine the evidence of TF–gene co-expression with evidence of the target gene’s association with phenotypic information, thereby reporting TF–gene regulatory relationships more relevant to the transcriptomic differences among phenotypic classes. Motivated by recent studies that have used summary statistics in place of original data to improve the computational efficiency and generalizability of the model to a wide range of data types^23,28–32^, InPheRNo also utilizes summary statistics (*p* values) to model these evidence, discussed above. The method is outlined in Fig. 1d and 1e and explained in Methods. We outline its main steps here.

Given the expression of genes and TFs across all samples, first a regression model is used to predict each gene’s expression as a weighted sum of TF expression values. This step uses the Elastic Net regression model^33^, which automatically selects a small number of candidate TFs regulating each gene. Next, an ordinary least squares (OLS) regression model is used to obtain a pseudo *p* value, reflecting the statistical relationship between each TF and that gene, in terms of their expression variation across all samples (see Supplementary Methods for reasons behind this two-step procedure to obtain pseudo *p* values). Note that both of the previous steps use multivariable regression techniques to relate a gene’s expression to the expression of a combination of TFs rather than one TF at a time. Separately, a *p* value of association between the gene’s expression and the phenotypic score is obtained using a suitable statistical test (the choice of which depends on the phenotypic variable, the data distribution, and potential confounders). This step allows for different types of phenotypic scores, including categorical labels with two or more values as well as numeric scores, to be incorporated into the method since the gene–phenotype relationship only needs to be encapsulated in a *p* value.

The two sets of *p* values from the above steps—one capturing TF–gene regulatory relationships and the other gene–phenotype associations—are then used as observed variables in a probabilistic graphical model (PGM) (and particularly a Bayesian Network^34^). The PGM has a latent Boolean variable for each TF–gene pair, indicating whether the TF regulates the gene so as to affect the phenotype. If this variable is “true”, the model expects to see evidence of the TF–gene pair being co-expressed and the gene’s expression being statistically associated with the phenotypic score. Posterior probabilities for these latent variables are then used to predict the edges of a phenotype-relevant TRN, and are estimated using Markov chain Monte Carlo (MCMC) algorithm (see Methods for more details).

It is worth mentioning that InPheRNo considers the simultaneous effect of multiple TFs on each gene in multiple steps of its pipeline. These include (1) utilization of a multivariable Elastic Net model to relate the expression of multiple TFs to the expression of the target gene in the TF selection step, (2) obtaining a pseudo *p* value for each TF–gene pair using a multivariable OLS model, which includes the expression of all selected TFs, and (3) the design of the PGM such that for each gene it models the relationship of observed data to the latent variables representing all selected TFs simultaneously.

### InPheRNo identifies cancer type-relevant TRNs in a pan-cancer study

**Fig. 2:**
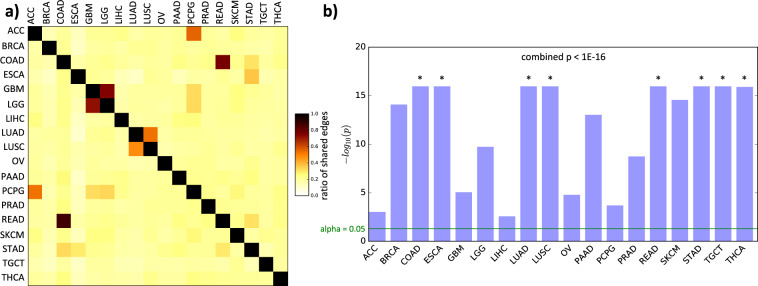
Characteristics of the cancer-relevant regulatory edges identified using TCGA data on 18 cancer types. **a** The heatmap shows the ratio of the shared regulatory edges between a pair of cancers to the total number of edges. More precisely, for any two cancers C_i_ and C_j_, the value in cell i, j shows the number of shared regulatory edges divided by the number of regulatory edges in C_i_. **b** The overlap between InPheRNo-identified TRNs for different cancers and global TRNs identified by TREG using ChIP-seq data. The bars represent −log_10_(*p*) of enrichment (hypergeometric test), truncated at 1E-16. The green line shows the threshold alpha = 0.05 and the symbol * is used for cases in which *p* < 1E-16. The combined *p* value is calculated using Fisher**’**s method. For the enrichment analysis using randomized degree-preserving networks and randomized degree distribution-preserving networks see Supplementary Data 3.

We applied InPheRNo to the gene expression profiles of 6357 primary tumor samples corresponding to 18 different cancer types from TCGA, downloaded from the Genomic Data Commons^35^ (see Table 1). For each cancer type, InPheRNo was used to reconstruct TRNs relevant specifically to that cancer type (as compared with all other types), setting the phenotypic label of each sample to be a Boolean variable representing whether the sample is from that cancer type or not, and using a two-sided *t* test to obtain gene–phenotype association *p* values. The cancer type-relevant TRNs thus obtained are provided in Supplementary Data 1 and the extent of shared regulatory edges between each pair of cancer types is shown in Fig. 2a. By and large, the TRNs are noted as being specific to each cancer type (average Jaccard coefficient of shared edges is at a relatively low value of 0.12), though the pairs (ACC, PCPG), (LGG, GBM), (LUAD, LUSC), and (COAD, READ) exhibit relatively large sharing of edges (average ratio of shared edges = 0.54, 0.73, 0.50, 0.82, respectively), partly owing to their shared tissues of origin. Also noticeable is the high degree of edge-sharing among gastro-intestinal cancers: STAD, COAD, READ, and ESCA (average ratio of shared edges = 0.36).

**Table 1:** Name, abbreviation, and number of samples for each cancer type used in this study.

Name of the cancer	Abbreviation	Number of samples
Adrenocortical carcinoma	ACC	79
Brain lower grade glioma	LGG	511
Breast invasive carcinoma	BRCA	1091
Colon adenocarcinoma	COAD	456
Esophageal carcinoma	ESCA	161
Glioblastoma multiforme	GBM	154
Liver hepatocellular carcinoma	LIHC	371
Lung adenocarcinoma	LUAD	513
Lung squamous cell carcinoma	LUSC	501
Ovarian serous cystadenocarcinoma	OV	374
Pancreatic adenocarcinoma	PAAD	177
Pheochromocytoma and paraganglioma	PCPG	178
Prostate adenocarcinoma	PRAD	495
Rectum adenocarcinoma	READ	166
Skin cutaneous melanoma	SKCM	103
Stomach adenocarcinoma	STAD	375
Testicular germ cell tumors	TGCT	150
Thyroid carcinoma	THCA	502

Owing to differences in tissues of origin of the studied cancer types, some of the edges identified as relevant to one cancer compared to others may reflect these tissue differences and not the cancers themselves. To address this and to better characterize cancer type-specific mechanisms, we additionally applied InPheRNo to gene expression profiles of 4388 normal tissue samples in the GTEx data portal^25^, corresponding to the 18 cancer types above (Supplementary Table 1 in Supplementary Information). The identified tissue-relevant TRNs (Supplementary Data 2) should enable us to distinguish between regulatory mechanisms in a normal tissue from regulatory mechanisms involved in a cancer, originating from that tissue, a direction we pursue later.

As a preliminary assessment of their accuracy, we sought to determine whether the identified cancer type-relevant TRN edges are enriched in independently identified TF–gene relationships using ChIP-seq data. Although the TRNs derived above are meant to be phenotype-relevant, they reflect regulatory relationships and are thus expected to be enriched in globally characterized regulatory edges, albeit to different degrees depending on the specific cancer type. We therefore used global TRNs (i.e., not cancer type-specific) reconstructed from ChIP-seq profiles of 166 TFs in 43 different cell lines from the ENCODE project, using the TREG method^36^ (see Methods for details). Figure 2b, Supplementary Fig. 2 (in Supplementary Information), and Supplementary Data 3 show the extent to which the cancer type-relevant TRN edges identified using InPheRNo are enriched for global TRN edges. We observed significant enrichments for every cancer type (using hypergeometric test, randomized degree-preserving test, and randomized degree distribution-preserving test), but to different degrees. Similarly, for all tissues except one, tissue-relevant regulatory edges obtained by applying InPheRNo on GTEx data are enriched in global regulatory edges (Supplementary Fig. 2 in Supplementary Information and Supplementary Data 3).

We noted a significant correlation between different cancer types and their corresponding normal tissues in terms of their enrichment for global TRN edges (Spearman’s rank correlation = 0.63, *p* = 4.8E-3 for the results obtained using the hypergeometric test). This suggests that some of the regulatory mechanisms identified from the TCGA data reflect the differences in regulatory mechanisms of the tissues of origin. To correct for this confounding effect, for each cancer we removed all the edges that were also present in the TRN identified for its corresponding normal tissue. In doing so, we augmented our approach of phenotype-relevant TRN reconstruction with the core idea of “differential network analysis” mentioned above, in the hope of achieving increased specificity to the cancer type. (Note that since the majority of cancer types considered in this study correspond to different tissue types, typical methods of removing confounders could not be used here). Depending on the cancer type, this procedure removed 7.0% (for READ) to 10.3% (for LUSC) of the identified edges (Supplementary Fig. 3 in Supplementary Information). The number of shared edges among cancer type-relevant TRNs of cancers originating from the same tissue (related to Fig. 2a) reduced upon correcting the confounding effect of the tissue of origin. However, this reduction was relatively small: 12.8% for LUSC and LUAD, 10.9% for GBM and LGG, 9.6% for ACC and PCPG, and 8.5% for COAD and READ. This suggests that the relatively high degree of edge-sharing among these pairs of cancers (Fig. 2b) cannot be simply explained by the regulatory mechanisms of their normal tissue of origin. The analyses reported in the rest of the manuscript correspond to these “tissue-corrected” cancer type-relevant TRNs (available in Supplementary Data 4).

### InPheRNo identifies breast cancer-relevant “driver” TFs, improving upon related methods

**Fig. 3:**
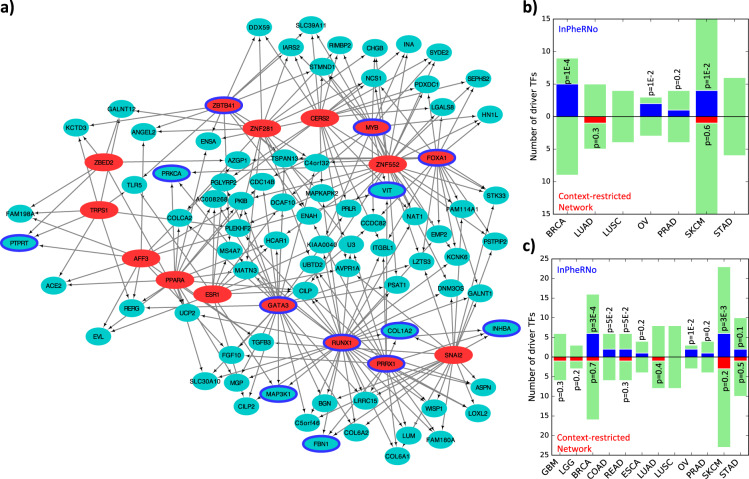
Driver TFs in cancer type-relevant TRNs reconstructed by InPheRNo. **a** A subnetwork of the BRCA-relevant TRN. The depicted subnetwork consists of the 15 TFs (red) with the most target genes, as well as genes (green) that are regulated by at least three of these TFs. Genes or TFs with a blue border represent BRCA drivers according to IntOGen and DriverDBv2. **b**–**c** Cancer specificity of InPheRNo in identifying driver TFs (using IntOGen) compared to the context-restricted network analysis (for results corresponding to other methods see Supplementary Figs. 4–8). For each cancer type, 100 TFs with the most number of identified target genes are selected and are compared with the set of driver TFs of that cancer that are drivers of at most *n*_*s*_ other cancers. Color green shows the total number of cancer-specific driver TFs in the IntOGen database, color blue corresponds to the number of cancer-specific driver TFs identified by InPheRNo and red represents driver TFs identified using context-restricted network analysis. Only cancers that had more than one known cancer-specific driver TF are used for the analysis. The *p* values are calculated using a hypergeometric test. **b** Results corresponding to *n*_*s*_ = 2. **c** Results corresponding to *n*_*s*_ = 3.

It is challenging to assess the accuracy and cancer-relevance of predicted TF–gene relationships on a global scale. However, TFs with many target genes in our cancer type-relevant TRNs are expected to play important roles in different traits of cancer, and existing databases of cancer drivers may therefore help us evaluate the TRNs. Accordingly, we examined the concordance between key TFs identified in the cancer type-relevant TRNs above and known driver TFs for that cancer as cataloged in the DriverDBv2^37^ and IntOGen^38^ databases. We focused on breast cancer (BRCA) given the relatively extensive knowledge of driver genes for it. We examined the BRCA-relevant TRN reconstructed using InPheRNo (Fig. 3a) and identified 15 TFs with most targets (Table 2) in this network. This set included six BRCA-driver TFs (RUNX1, GATA3, MYB, FOXA1, ZBTB41, PRRX1) according to DriverDBv2^37^ (*p* = 1.2E-4, hypergeometric test) and four (RUNX1, GATA3, MYB, FOXA1) according to IntOGen^38^ (*p* = 2.3E-4, hypergeometric test). To assess if the InPheRNo TRNs exhibit an improved ability to reveal driver TFs, we repeated the above evaluations with results from six related approaches (“baselines”, see Methods and Supplementary Fig. 1 for details), as outlined below.

**Table 2:** Top 15 TFs identified using InPheRNo and the evidence for their role in breast cancer.

Transcription factors	Percent of target genes	Evidence
RUNX1	6.7%	Driver (IntOGen, DriverDBv2)
ZNF552	4.9%	Modest Literature Support
GATA3	4.6%	Driver (IntOGen, DriverDBv2)
MYB	4.4%	Driver (IntOGen, DriverDBv2)
CERS2	4.3%	Strong Literature Support
FOXA1	4.3%	Driver (IntOGen, DriverDBv2)
SLUG	4.2%	Strong Literature Support
AFF3	3.8%	Modest Literature Support
ZNF281	3.7%	Modest Literature Support
ZBED2	3.7%	No evidence found
ZBTB41	3.7%	Driver (DriverDBv2)
PRRX1	3.7%	Driver (DriverDBv2)
TRPS1	3.6%	Strong Literature Support
ESR1	3.5%	Strong Literature Support
PPARA	3.4%	Strong Literature Support

The TFs are ranked based on the number of their cancer-relevant target genes. The second column shows the percent of the considered genes that each TF regulates, and the third column shows the type of evidence supporting each TF. Although we labeled the literature support for each TF as “modest” or “strong” based on our judgment, the evidence and its reference are provided in the main text for clarification and completion.

In the first baseline, we constructed a “context-restricted” TRN using only breast cancer samples, mimicking similar approaches in the literature^8–11^. We modeled each gene’s expression in terms of the expression values of all TFs, via multivariable regression. We adopted the Elastic Net algorithm for this purpose, as in the first step of InPheRNo, obtaining a small number of TFs regulating each gene (see Methods), and ranked TFs by the number of target genes. The top 15 TFs identified using this approach included no BRCA-driver TF according to either of the two databases. In the second baseline, we used DiNA^12–16^ to identify edges that are present in the TRN reconstructed using BRCA samples and not present in the TRN reconstructed using samples of other cancers pooled together (see Methods). (TRN reconstruction relied on the Elastic Net algorithm, exactly as in the first baseline.) The set of 15 TFs with the greatest number of target genes using this approach contained only one known BRCA-driver TF according to DriverDBv2 and none according to IntOGen. The third baseline was a “context-specific” TRN^17–19^ reconstructed by relating the expression of differentially expressed genes to the expression of TFs (see Methods). The set of top 15 TFs identified using this approach did not include any BRCA-driver TFs according to any of the two databases. The fourth baseline involved identifying TFs whose expression had the most significant difference between samples of the breast cancer compared with samples of other cancers (Welch’s *t* test). (That is, no TRN reconstruction was performed.) The set of 15 TFs identified using this approach did not contain any driver TFs according to IntOGen or DriverDBv2. For the fifth baseline, we used the MRA tool^20,39^ to identify 15 master regulators of BRCA. This analysis identified only one driver TF according to DriverDBv2 and none according to IntOGen. For the sixth baseline, we used an approach based on Fisher’s method to combine the *p* value of association between a gene’s expression and the phenotype with the *p* value of Pearson’s correlation between expression of that gene and the expression of a TF (see Methods for details). This method, which can be considered a simplified version of InPheRNo, has the benefit of reconstructing phenotype-relevant co-expression networks efficiently, but does not allow us to simultaneously model the effect of multiple TFs on each gene. In spite of this shortcoming, this method, henceforth called “simplified-InPheRNo”, outperformed all other methods except for InPheRNo in identifying BRCA-driver TFs: the list of 15 TFs with the greatest number of target genes included four driver genes according to either database. The top TFs identified using these different methods are provided in Supplementary Data 5.

We noted above that six of the 15 key TFs of the BRCA-specific TRN determined by InPheRNo are known driver TFs. We mined the literature and found strong evidence for the role of five additional TFs (from the remaining nine) in BRCA; see Table 2. For instance, ESR1 encodes estrogen receptor alpha and its role in the development, progress, and drug resistance of breast cancer is well documented^40–42^. CERS2 is a ceramide synthase and suppresses breast tumor cell invasion and enhances chemosensitivity of breast cancer cells^43,44^. In addition, the low expression of this gene is associated with poor prognosis in breast cancer^44^. SLUG is a TF involved in epithelial to mesenchymal transition (EMT) and is known to promote breast cancer progression and invasion^45–47^. We recently showed that this TF (along with FOXA1, another TF identified by InPheRNo, Table 2) is a biomarker of metastatic subtypes of breast cancer^48^. TRPS1 is a transcription repressor of GATA-regulated genes, which promotes EMT in breast cancer and its expression is associated with clinical outcome in this cancer^49,50^. The activation of PPARA has been shown to promote proliferation in human breast cancer and its genetic polymorphism has been linked to an increase in the odds of postmenopausal breast cancer^51,52^.

In addition to the above five, three other TFs among the top 15 identified by InPheRNo have modest literature support for a role in BRCA development: AFF3 is a nuclear transcriptional activator, which is abnormally expressed in some cases of breast cancer and has been suggested as a proto-oncogene^53,54^. ZNF281 is a transcriptional repressor involved in EMT that is upregulated in colon and breast cancer and has been suggested to promote these cancers^55,56^. In addition, ZNF552 has been suggested as a regulator of genetic risk of breast cancer and its regulons have shown to be enriched in genes associated with risk loci identified using a combination of GWAS and eQTL analysis^57^. Taken together, these results suggest that InPheRNo can accurately identify regulatory mechanisms (in this case, major TFs) involved in breast cancer.

In addition to the breast cancer-relevant TRN above, we used InPheRNo to reconstruct PAM50 subtype-relevant TRNs in breast cancer (see Supplementary Methods for details). In this application, the phenotypic label of each sample reflects its PAM50^26^ subtype (a categorical variable with five categories), illustrating the applicability of InPheRNo to different types of phenotypic labels/scores. The reconstructed TRN (Supplementary Data 6) implicated several key TFs well documented to be involved in different subtypes of BRCA (see Supplementary Methods and Supplementary Data 7). Particularly, among the top 14 TFs, four (SR1, FOXA1, FOXC1, MYBL2) were among the TFs of the PAM50 gene signature (*p* = 1.46E-7, hypergeometric test), further indicating their role in regulatory mechanisms of breast cancer subtypes.

### Driver TFs identified by InPheRNo are specific to respective cancer types

We next asked if the key TFs (those with most target genes) in InPheRNo-derived TRNs are specific to their respective cancer types, as this is an important criterion for phenotype-relevant TRN reconstruction. We obtained a list of driver TFs for each cancer from IntOGen, and retained only those known drivers that were not annotated as drivers for more than *n*_*s*_ = 2 other cancer types (to ensure cancer specificity). We then compared these cancer type-specific drivers, whose counts ranged from 0 to 15, depending on the cancer, to the top 100 TFs identified for that cancer using InPheRNo (Supplementary Data 5). Of the seven cancer types that had more than one known driver TF specific to them, three cancers (BRCA, OV, and SKCM) showed a significant (alpha = 0.05) enrichment between InPheRNo-identified TFs and known cancer type-specific drivers, with an overall combined *p* value (Fisher’s method) of *p* = 2.5E-4 (Fig. 3b). However, repeating the above procedure with key TFs identified by context-restricted network analysis, DiNA, MRA, context-specific network analysis, or based on differential expression did not yield significant enrichment for cancer type-specific drivers in any of these seven cases (Fig. 3b and Supplementary Fig. 4 in Supplementary Information). Key TFs of TRNs determined by simplified-InPheRNo were significantly enriched for known drivers in two cases (Supplementary Fig. 4 in Supplementary Information).

Similar observations were made when using a slightly relaxed definition of a cancer type-specific driver TF: as a known driver of one cancer type that is not a known driver for more than *n*_*s*_ = 3 other cancer types (Fig. 3c, Supplementary Fig. 5 in Supplementary Information). For the 12 cancer types where two or more such cancer type-specific drivers are known, InPheRNo-identified key TFs showed the highest enrichment for those drivers (combined *p* = 6.2E-5) compared with simplified-InPheRNo (combined *p* = 6.6E-4), top differentially expressed TFs (combined *p* = 0.62), differential network analysis (combined *p* = 0.64), context-restricted analysis (combined *p* = 0.92), MRA (combined *p* = 0.99), and context-specific analysis (combined *p* = 0.99). Although the above analyses were performed using driver TF annotations from IntOGen, similar analysis using driver genes in DriverDBv2 also confirmed the conclusion that InPheRNo has a high specificity in identifying regulatory mechanisms involved in each cancer, especially when compared with related approaches (Supplementary Figs. 6–8 in Supplementary Information). We believe this relatively high specificity of InPheRNo arises from the explicit and quantitative incorporation of phenotypic labels into its statistical model.

### Gene expression signatures based on InPheRNo TRNs are predictive of patient survival

**Fig. 4:**
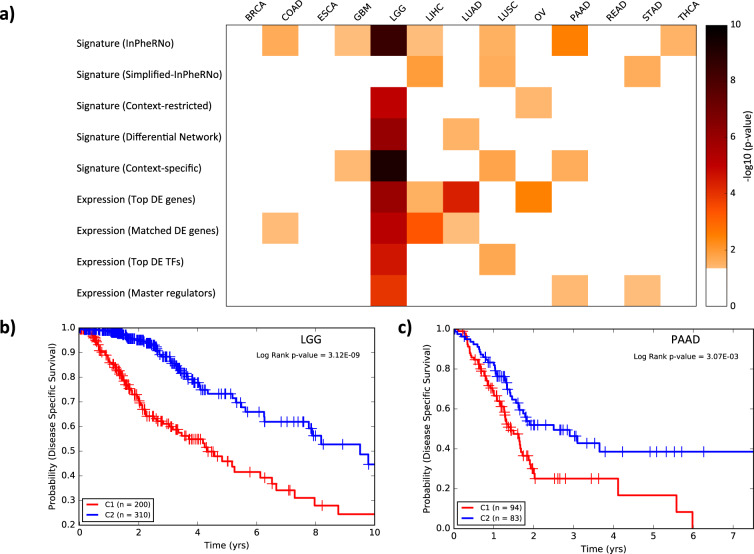
Survival analysis for samples of different cancers clustered using different approaches. **a** The heatmap shows the performance of different approaches used for clustering of samples. Samples of each cancer are clustered into two groups and each cell in the heatmap represents −log_10_(*p*) (obtained using a log-rank test) of the significance of the difference between survival probabilities of the two clusters. for clarity, cases in which the *p* value was larger than 0.05 are shown as white. **b**–**c** Kaplan–Meier analysis for two clusters obtained by the gene expression signature formed by the top five TFs and their target genes, as identified by InPheRNo for LGG **b** and PAAD **c** cancer types.

Gene expression signature analysis is a widely used approach in analyzing and subtyping cancer samples, with great potential for improving prognosis and treatment^58,59^. We hypothesized that since InPheRNo identifies cancer type-relevant regulatory mechanisms, the resulting TRNs can be used to form gene expression signatures that are more predictive of patient survival than signatures formed using differential expression analysis, one of the most widely used approaches for forming gene expression signatures^59^. It has been previously suggested that the activity of a TF is better reflected in the activities of its targets taken together than its own expression^20^. Therefore, we formed a gene expression signature for each TF, reflecting the expression of the TF as well as the activity levels of its targets in the InPherRNo-derived TRN, while considering the predicted strength and direction of regulation for each gene (see Methods for details). For each cancer type, we formed a sample by signature matrix (five columns corresponding to the five signatures of the key TFs with the greatest number of target genes in the corresponding TRN) and clustered patient tumor samples (the rows) into two groups (hierarchical clustering). (See Supplementary Methods and Supplementary Table 2 for how the number of TFs influences results). We used Kaplan–Meier survival analysis to determine whether these two clusters show distinct survival behavior, limiting our analysis to cancers with >150 samples and more than ten incidents of death. Out of the 13 cancers satisfying these conditions, the expression signatures classified samples into clusters of distinct survival (log-rank test, alpha = 0.05) for seven cancers (Fig. 4), with LGG having the smallest *p* value (*p* = 3.1 E-09).

We repeated the above survival analysis using gene expression signatures created from TRNs reconstructed by context-restricted analysis, DiNA, MRA, context-specific analysis, and simplified-InPheRNo, which resulted in one to four significant cases (Fig. 4a, Supplementary Table 3 in Supplementary Information), in contrast to the seven noted above for InPheRNo. Similarly, clustering based on top five most significantly differentially expressed genes or TFs resulted in four and two significant cases, respectively. The results did not improve when we used the same number of differentially expressed genes as was used in forming InPheRNo’s gene signature, yielding only four significant cases. These results show that taking into account the phenotype-relevant regulatory mechanisms identified by InPheRNo in developing gene expression signatures may improve the performance of gene signature analysis and prediction of survival.

**Fig. 5:**
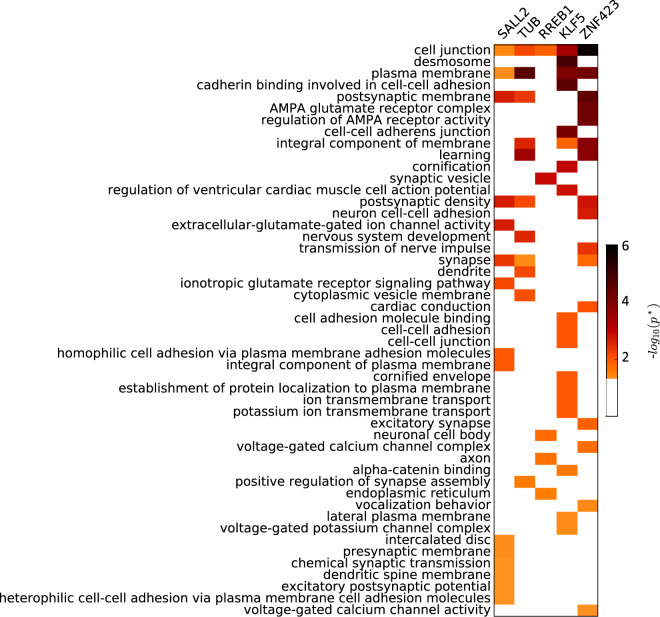
Functional annotation of top five TFs and their targets identified using InPheRNo for LGG. The heatmap shows the Benjamini–Hochberg corrected GO enrichment false discovery rates (FDR). For clarity, cases in which the FDR was larger than 0.05 are shown as white. The GO terms are sorted based on the smallest FDR in any of the five gene sets.

Given the observation that the gene expression signature formed using the InPheRNo-identified TRN for Lower Grade Glioma (LGG) can accurately predict patients’ prognosis (Fig. 4b), we sought to determine the functional characteristics of these genes. To this end, we performed gene ontology (GO) enrichment analysis using KnowEnG analytical platform^60,61^ for each of the five TFs (identified using InPheRNo and used earlier for survival analysis) and their targets, one TF at a time. Overall, 49 GO terms with size larger or equal to ten were enriched (Fisher’s exact test, Benjamini–Hochberg corrected false discovery rate p^*^ < 0.05) for these gene sets (Fig. 5 and Supplementary Data 8). Out of these GO terms, 21 were related to the nervous system, neurotransmission, and neurogenesis. On the other hand, 12 other terms were related to cell junction, which plays an important role in the invasion-metastasis cascade in various cancers including gliomas^62,63^. These results support our expectation that both regulatory mechanisms specific to nervous system as well as more general cancer-related mechanisms are involved in the development and progress of LGG.

We repeated the GO enrichment analysis above for top five TFs and their targets implicated by InPheRNo in PAAD, for which InPheRNo gene expression signature outperformed all other approaches in the survival analysis (Fig. 4a). We observed the enrichment of several key GO terms (Supplementary Data 9). For example, desmosomes (FDR = 0.034), which are intercellular adhesion complexes, have been shown to play important roles in tumor invasion in mouse models of pancreatic neuroendocrine cancer^64^. Moreover, their role in various traits of cancers such as tumor suppression has been documented in various cancers^65–67^. Several GO terms were related to collagen, which have been shown to promote metastasis^68^, cell growth and proliferation^69^, and cell survival^70^ in pancreatic cancer. Many GO terms were related to extracellular matrix, which has been shown to promote cell survival, cell proliferation, and metastasis of pancreatic cancer cells in various studies^71,72^. Taken together, these results show the ability of InPheRNo to identify key biological processes involved in different cancer types such as LGG and PAAD.

## Discussion

TRNs provide an important and popular framework for better understanding a cell’s regulatory mechanisms, leading to phenotypic conditions. However, to the best of our knowledge TRN reconstruction methods today do not incorporate phenotypic information adequately or at all. As such, the reconstructed networks may be limited in pinpointing regulatory mechanisms most related to a phenotype under investigation, and often necessitate a follow-up step that filters for phenotype relevance. For example, a recent study of gene expression changes underlying Huntington’s disease (HD)^73^ reconstructed a TRN specific to the mouse striatum and then short-listed TFs whose predicted targets were enriched in genes differentially expressed in HD mouse models. In another study, gene expression profiles of TFs and putative target genes were used to reconstruct a context-restricted TRN for breast cancer (using only breast cancer samples), and then a list of breast cancer-relevant TFs (called “risk-TFs”) whose regulons were enriched in risk loci were short-listed^57^. In the aforementioned study^57^, GWAS and eQTL analyses were used to define risk loci and relate them to the regulon of each TF. Such previous attempts to augment TRN reconstruction with phenotypic data motivated us to develop a systematic approach to incorporate information about the phenotype directly into TRN reconstruction.

In this study, we developed InPheRNo to reconstruct phenotype-relevant TRNs and utilized it to identify regulatory interactions that differentiate one cancer type from others while correcting for the confounding effect of tissues of origin. InPheRNo is based on a carefully designed PGM, which is key to combining TF–gene expression correlations with gene–phenotype associations. The conditional distributions of the PGM model the summary statistics of gene–phenotype and TF–gene associations, providing a succinct and efficient approach for data integration to identify phenotype-relevant regulatory relationships. The method is broadly applicable since it learns regulatory relationships from expression data alone and does not impose any restriction on the type of phenotype under investigation—the phenotype may be binary, categorical or even continuous-valued, and any appropriate statistical method for testing its association with a gene’s expression may be used in InPheRNo. Unlike several other methods that rely on the regulatory relationship of one TF–gene pair at a time, InPheRNo considers the effect of multiple TFs on each gene in the reconstruction procedure, at the time of selecting candidate TFs as well as in training the PGM. Finally, using posterior probabilities obtained from the PGM, InPheRNo provides a score representing the confidence for the identified phenotype-relevant regulatory edges.

In designing InPheRNo’s pipeline, we made the choice to first perform a feature selection step (using Elastic Net) and only use the selected TFs in the PGM. First and foremost, this was done to reduce the computational complexity, both by reducing the number of candidate TFs and also by summarizing the expression profiles of genes and TFs using summary statistics. Several previous studies have successfully used summary statistics (and particularly *p* values) for similar reasons^23,28–32^. Second, modeling summary statistics instead of the full gene expression data enables integration of other regulatory evidence (captured through data types other than transcriptomic, if available) in the PGM with a relative ease.

One important consideration when using InPheRNo, is the number of samples. As InPheRNo is based on modeling of summary statistics obtained from gene–phenotype and gene–TF associations, similar requirements on the minimum number of samples for those analyses should be also considered here^74–76^. However, two features of InPheRNo enable it to handle a small number of samples better than traditional co-expression analysis. First, it utilizes Elastic Net (as part of the pipeline), whose regularization terms can overcome some limitations of the small sample size by imposing sparsity criterion. Second, as its PGM models the distribution of the *p* values instead of relying on whether such *p* value are significant or not (i.e., instead of thresholding them) it is more robust towards small samples sizes.

As there are no rigorously validated metazoan TRNs to benchmark against, we evaluated the predicted TRNs indirectly through key TFs and gene expression signatures derived from them, and showed clear improvement over several related strategies. Our results showed that the TFs with many cancer type-relevant targets are potential cancer driver TFs and may suggest novel drug targets or provide new insights, regarding the development and progress of cancer. Our results also suggest a powerful approach for subtyping of cancer patients using gene expression signatures: while most approaches developed for this task do not take into account the regulatory interactions among genes, our survival analysis suggests that cancer type-relevant TRNs can improve the predicting power of gene expression signatures.

In spite of the success of the InPheRNo-based gene signatures in differentiating between patients with poor and good prognosis for the majority of cancer types, in some cases, e.g., BRCA, this method did not result in groups with significantly different survival probability, despite the existence of BRCA-driver TFs in the signature. This lack of success may partially be owing to the fact that we clustered samples of each cancer type into two clusters, whereas these cancer types may include more than two subtypes, as is the case in BRCA^26^. However, since in most cancer types a definite number for the cancer subtypes is not yet established, we preferred to keep the number of clusters equal to two. A more in-depth analysis of subtype discovery and survival analysis using InPheRNo-derived TRNs is left for future work.

We would like to emphasize that in this study, we focused only on transcriptomic data, owing to the availability of this data type in many domains, including domains outside of cancer research, and lack of other important data types such as ChIP-seq data in these domains. Even in the area of cancer research, in which large databases of ChIP-seq tracks (such as ENCODE) corresponding to various cancer cell lines are available, the datasets are extremely biased toward a small fraction of well-studied TFs (for example only ~10% of all TFs are studied in ENCODE). As a result, including these data sets may significantly bias the analysis towards this small fraction of TFs. In addition, matched gene expression and ChIP-seq data for tumor samples are rarely available and combining these data types from different sources and different samples, in itself a significant challenge, will require substantial effort in the future.

We believe that including additional types of regulatory evidence (especially those representing “cis” mechanisms such as TF motifs and chromatin state changes) in the phenotype-relevant TRN reconstruction procedure is an important and essential future direction for improving InPheRNo. This is especially true considering that many efforts are under way to generate large datasets containing matching transcriptomic, genomic, epigenomic and phenotypic profiles of patients^77–79^. One way to achieve this goal might be to include different regulatory evidence as new observed variables in the PGM used in InPheRNo. Another alternative is to use cis-regulatory evidences to construct an initial network that is used as a “prior” for Bayesian analysis of expression data, as has been demonstrated before^80^. Future investigations should focus on these avenues of integrating multi-omics data into the InPheRNo model.

## Methods

### Inference of Phenotype-relevant Regulatory Networks (InPheRNo)

InPheRNo (Figs. 1d, e) is a new computational method for reconstructing phenotype-relevant TRNs. At its core, InPheRNo utilizes a carefully designed PGM (and more specifically a Bayesian Network^34^) (Supplementary Fig. 9 in Supplementary Information) to systematically combine the information on the significance of gene–phenotype associations with the information on the significance of gene–TF associations to obtain a phenotype-relevant TRN. In addition, InPheRNo takes into account the simultaneous effect of multiple TFs on each gene.

As input, InPheRNo accepts a matrix of gene and TF expression data (gene and TFs x samples), a list of TFs and a vector ***p*** that records the *p* value of association between the expression of each gene and the variation in the phenotypic scores/labels of samples (obtained using a suitable statistical test depending on the type of phenotype), as depicted in Fig. 1d. We assume that the expression matrix is properly normalized in advance, such that the distribution of each gene and TF across all samples approximately follows a standard Normal distribution (see Supplementary Methods). Using the list of TFs, the gene expression matrix is divided into a matrix ***X*** of TF expression data (TFs x samples) and a matrix ***Y*** of gene expression data (genes x samples).

In order to obtain a measure of significance for the association between each gene–TF pair, while considering the influence of other TFs on the gene of interest, we used a two-step procedure. First, we used Elastic Net, a linear multivariable regression algorithm that imposes sparsity using regularization, to identify a set of *m*_*j*_ candidate TFs for each gene $j\left( {j = 1,\,2, \cdots ,n} \right)$. The Elastic Net step of InPheRNo was implemented using the function ElasticNetCV in the scikit-learn library (version 0.18.1) for python^81^. This library implements Elastic Net by minimizing the objective function,

1 $$\frac{1}{{2n}}\left\| {{\boldsymbol{y}} - {\boldsymbol{Xw}}} \right\|_2^2\, +\, \mu \rho \left\| {\boldsymbol{w}} \right\|_1\, +\, \frac{1}{2}\mu \left( {1 - \rho } \right)\left\| {\boldsymbol{w}} \right\|_2^2,$$

where *n* is the number of samples, ***y*** is the response vector, ***X*** is the feature matrix, ***w*** is the unknown vector of coefficients, and *μ* and *ρ* are hyperparameters. In this model, we used the TF expression matrix ***X*** as the feature matrix and the expression profile ***y***_*j*_ of each gene as the response vector. The hyperparameter *μ* was chosen using cross-validation by iteratively fitting the model along a regularization path. We used the default value of the library for *ρ* (*ρ* = 0.5). In addition, we imposed the constraint that the maximum number of nonzero coefficients in the learnt model should be at most equal to *m*_max_ = 15, to reduce the computational complexity of the following steps and impose the prior knowledge that only a few TFs regulate each gene. Note that imposing an upper limit on the number of regulators of a gene has been previously used for various reasons including the reasons above^82–84^. It is important to note that *m*_max_ is only an upper limit, and the best number of TFs for each gene is obtained by Elastic Net and the following PGM.

Next, for each gene *j* we formed a matrix ***X****j* representing the expression of the *m*_*j*_ selected TFs across different samples. Then, we used ***X****j* as the feature matrix in a multivariable OLS regression model to relate the expression of the identified TFs to the expression of the gene ***y****j* (the response vector) and calculated a pseudo -value *π*_*i,j*_ (using the OLS model), reflecting the conditional effect of the TF $i\left( {i = 1,\,2, \cdots ,m_j} \right)$ on gene *j*. Using the OLS regression model is a necessary step, since current approaches for calculating the *p* value of feature-response associations in regularized regression models require assumptions that are not satisfied in this application^85^ or require resampling or data splitting that reduces the statistical power^86,87^ (see Supplementary Methods for a discussion on these alternative methods and their assumptions). It is important to note that *π*_*i,j*_ is only a “true” *p* value for the second step of this procedure, but does not satisfy all the characteristics of a *p* value for the two-step procedure (see Supplementary Information for simulation results). More precisely, under the Null hypothesis that TF *i* is not associated with gene *j*, the distribution of *π*_*i,j*_ is not uniform (a characteristic of a true *p* value), but instead is biased towards small values (see Supplementary Figs. 10–12 in Supplementary Information). The reason for this bias is that in the first step, Elastic Net selects TFs whose expression are associated with the expression of gene *j* and the second step is thus likely to assign a small *p* value to them. This is an important consideration, since it affects how we model the conditional distributions of *π*_*i,j*_s in the PGM described below.

The two sets of *p* values—one capturing TF–gene regulatory relationships (denoted as *π*_*i,j*_) and the other gene–phenotype associations (denoted as *P*_*j*_ and provided in vector ***p***)—are used as observed variables in a PGM (Supplementary Fig. 8) that has binary latent variables *T*_*i,j*_ reflecting the role that each putative TF–gene interaction plays in phenotype variation. More precisely, *T*_*i,j*_ = 1 implies that TF *i* regulates gene *j* so as to affect the phenotype, and *T*_*i,j*_ = 0 indicates its logical complement. We modeled the prior distribution of this random variable as *T*_*i,j*_ ∼ *Bernoulli*(*γ*). The posterior probabilities of *T*_*i,j*_s obtained from this PGM can be used to form the phenotype-relevant TRN (as described below).

As depicted in Fig. 1e and Supplementary Fig. 9 (in Supplementary Information), InPheRNo uses a directed acyclic graph (DAG) to model the relationship between the latent variables and the observed variables. The topology of this DAG represents the idea that the value of *T*_*i,j*_ has a causal effect on the distributions of observed variables *P*_*j*_s and *π*_*i,j*_. Since each *P*_*j*_ represents a “true” *p* value, it follows a uniform distribution under the Null hypothesis that “expression of gene *j* is not associated with the phenotypic variation”, which is the scenario where gene *j* does not mediate the influence of any of its putative regulators on the phenotype. In other words, if $T_{1,j} = T_{2,j} = \cdots = T_{m_j,j} = 0$, then *P*_*j*_ ∼ *Unif* (0,1). On the other hand, if any of the *T*_*i,j*_s is equal to 1, the definition of *T*_*i,j*_ implies that gene *j* is associated with the phenotype (the alternative hypothesis). Following the approach in Hanson et al.^23^ who successfully used a Beta distribution to model the distribution of *p* values when they are biased towards small values, we used a *Beta* (*α*, *β*) distribution to model the distribution of these variables under the alternative hypothesis. By fixing *β* = 1 and limiting the value of *α* in the range 0 < *α* ≤ 1, we can obtain a wide range of distributions with different degrees of bias towards small values with the smallest bias when *α* = 1 (equivalent to a uniform distribution) and an increasing degree of bias as *α* approaches 0 (see Supplementary Fig. 13 in Supplementary Information). Thus, the conditional distribution of *P*_*j*_ given the value of its parent nodes in the DAG can be modeled as

2 $$P_j \sim \left\{ {\begin{array}{*{20}{l}} {Unif\left( {0,\,1} \right)} \hfill & {{\mathrm{if}}\,T_{1,j} = T_{2,j} = \cdots = T_{m_j,j} = 0} \hfill \\ {Beta\left( {\alpha = \alpha ^{\prime},\,\beta = 1} \right)} \hfill & {{\mathrm{otherwise}}} \hfill \end{array}} \right.$$

where $\alpha^ {\prime},\,0\, < \,\alpha^ {\prime} \le 1,$ is a parameter controlling the degree of bias of the Beta distribution towards small values. In our analyses, we estimated *α*’ by fitting a mixture of a uniform and a Beta distribution to the histogram of *P*_*j*_s for all genes, prior to training the PGM. Note that modeling the conditional distribution of each *P*_*j*_ based on the values of *T*_*i,j*_s (for all values of *i*) allows us to capture the influence of multiple TFs on the value of observed variables, and hence on the phenotype-relevant regulation of the genes.

As mentioned earlier, the pseudo *p* values *π*_*i,j*_s obtained using the two-step procedure are biased towards small values even when TF *i* is not a regulator of gene *j*. As a result, similar to the case with *P*_*j*_s, we can use two distributions $Beta\left( {\alpha = \alpha _{1j},\,\beta = 1} \right)$ and $Beta\left( {\alpha = \alpha _{0j},\,\beta = 1} \right)$ to model the distribution of *π*_*i,j*_s when TF *i* regulates gene *j* and when it does not, respectively. However, in order to differentiate between the aforementioned scenarios, we need to impose a restriction on the parameters of these two distributions relative to each other. We hypothesized that the bias towards small values is larger when TF *i* is a regulator of gene *j* compared with when it is not. Intuitively, this can be justified as follows: assuming a linear relationship between the expression of a gene and its regulators, the main reasons for existence of false-positive candidate TFs identified using Elastic Net are the high dimensionality of the data (more features compared with samples), existence of noise in the data and a lack of prior knowledge on the number of regulators of each gene. As a result, even when some false positives are identified using Elastic Net, most of the variance of the gene’s expression is expected to be explained using the expression of the true positive TFs. As a result, the expression of the true positive TFs will have a more significant association with the gene’s expression in an OLS model. We used extensive simulation analysis under different setups and confirmed the intuition above (Supplementary Table 4 and Supplementary Figs. 10–12 in Supplementary Information). As a result, we modeled the prior distribution of these unknown parameters according to $\alpha _{0j}\sim Unif\left( {0.5,\,1} \right)$ and $\alpha _{1j}\sim Unif\left( {0,\,0.5} \right)$, to ensure that *α*_0*j*_ > *α*_1_j and a more significant bias towards small values exists when TF *i* is a regulator of gene *j* (see Supplementary Fig. 13). To model the conditional distribution of *π*_*i,j*_ given its parents, we note that one implication of *T*_*i,j*_ = 1 is that TF *i* regulates gene *j*. On the other hand, if *T*_*i,j*_ = 0, either TF *i* does not regulate gene *j* or TF *i* regulates gene *j* but gene *j* is not associated with the phenotype. Consequently, we used the following model

3 $$\pi _{i,j} \sim \left\{ {\begin{array}{*{20}{l}} {Beta\left( {\alpha = \alpha _{1j},\,\beta = 1} \right)} \hfill & {{\mathrm{if}}\,T_{i,j} = 1} \hfill \\ {r_jBeta\left( {\alpha = \alpha _{1j},\,\beta = 1} \right) + \left( {1 - r_j} \right)Beta\left( {\alpha = \alpha _{0j},\,\beta = 1} \right)} \hfill & {{\mathrm{if}}\,T_{i,j} = 0,} \hfill \end{array}} \right.$$

where *r*_*j*_ is an unknown mixing parameter representing the probability that TF *i* regulates gene *j* but gene *j* is not associated with the phenotype. We assigned a prior distribution of $r_j\sim Unif\left( {0,1} \right)$ to this parameter (reflecting lack of prior knowledge).

We used a Markov chain Monte Carlo (MCMC) method using the PyMC python module^88^ to infer the unknown parameters and learn empirical posterior probabilities for *T*_*i,j*_s. As some of the solutions of the MCMC may converge to local optima, to alleviate their effect we ran the MCMC procedure 100 times with different random initializations and obtained an average posterior probability for each *T*_*i,j*_. These average values were then minmax normalized and an appropriate threshold was used to identify phenotype-relevant regulatory edges (we used a threshold of 0.5). Since several parameters can be configured by the user, for the default values, which were used in the pan-cancer analysis as well as the method used for hyperparameter selection see Supplementary Methods (in Supplementary Information).

Supplementary Methods and Supplementary Table 5 provide details on robustness analysis and false-positive analysis of InPheRNo, demonstrating different properties of this approach.

### Data collection and normalization

We downloaded a list of 1544 human TFs from AnimalTFDB^89^. Gene (including TF) expression profiles of 6357 cancer patients corresponding to 18 different cancer types in TCGA were downloaded from the Genomic Data Commons^35^. Similarly, the gene expression profiles of 4388 normal tissue samples corresponding to these 18 cancer types (version V6p) were downloaded from the GTEx data portal (www.gtexportal.org). To normalize the FPKM (TCGA) and RPKM (GTEx) values we used an approach similar to the guideline described in the GTEx data portal for analyzing gene expression corresponding to version V6p (https://gtexportal.org/home/documentationPage). The expression profile of each sample was normalized in two ways: for the analyses that involved expression of all samples (across different cancer or tissue types), a pan-cancer (pan-tissue) normalization was performed, whereas for the analyses that required samples of one cancer (tissue) type, a cancer (tissue)-specific normalization was performed (see Supplementary Methods in Supplementary Information).

For the comparison of the reconstructed networks using InPheRNo with a global (cancer-agnostic) TRN, we downloaded “ENCODE TREG binding profiles” from http://eh3.uc.edu/treg, which include the binding probabilities assigned to each (TF, gene) by TREG for 43 different cell lines, using only ChIP-seq profiles of the cell lines for these TFs. We then selected edges with “probability” larger than 0.5 and formed their union over all cell lines to obtain a global TRN.

We obtained from IntOGen^38^ (www.intogen.org) a list of driver TFs that are identified based on mutations, gene fusions, and copy number alterations. We then combined the driver lists for each of these three data types into one list for each cancer. We also obtained a list of cancer driver genes from DriverDBv2^37^ http://driverdb.tms.cmu.edu.tw/driverdbv2, selecting driver genes that were identified by at least two different methods.

### Related baseline approaches for network reconstruction

We used several related approaches as comparators for InPheRNo. The first four are methods for including information on phenotypic labels of samples in the TRN reconstruction procedure. However, the last one (master regulator analysis^20^) does not construct a TRN, but identifies key TFs related to the phenotype. Although the ultimate goal of this method is different from InPheRNo, including it in our analysis provides further insight regarding the performance of InPheRNo.

1. *Simiplified-InPheRNo:* to obtain cancer type-relevant networks using simplified-InPheRNo, we used the Pearson’s correlation to obtain the *p* values of TF–gene associations and a two-sided *t* test to obtain the *p* values of gene–phenotype associations differentiating one cancer type from other types of cancer. Next, for each (gene, TF, phenotype) triplet, we used Fisher’s method to combine the two *p* values. Then for each cancer type, edges with smallest *p* values were selected such that the number of edges in the reconstructed network would be equal to the number of edges identified by InPheRNo (for a fair comparison). We performed this analysis for each cancer type using TCGA data and each tissue type using GTEx data and used the same approach in InPheRNo to remove the confounding effect of tissues of origin.
2. *Context-restricted TRN reconstruction:* this approach (Supplementary Fig. 1a in Supplementary Information) refers to the family of methods that restrict the analysis to samples representing a particular biological context (e.g., a tissue type^8,9^ or a cancer type^10,11^) and exclude the samples corresponding to other contexts. Since any TRN reconstruction algorithm based on gene expression data can be used in this framework we used Elastic Net^90–92^, which we have also used as the first step of InPheRNo, to ensure a fair comparison between InPheRNo and context-restricted network analysis. Details of choosing the hyperparameters of the Elastic Net using cross-validation are provided in the Supplementary Methods (in Supplementary Information). To obtain a context-restricted network for each cancer type, we used the expression profile of a gene across samples of that cancer type as the response vector and the expression of the TFs as the feature vectors in the Elastic Net model to identify TFs with nonzero coefficients for each gene. To ensure the fairness of comparisons, we focused on the same subset of genes that were utilized by InPheRNo.
3. *Differential network analysis (DiNA):* DiNA (Supplementary Fig. 1b in Supplementary Information) is another approach to relate TRNs to the phenotypic binary labels (e.g., case vs. control). In this approach, two context-restricted networks are reconstructed based on samples from each of two phenotypic classes, and a differential network is formed by comparing these two networks^12–16^. To perform DiNA, we used the context-restricted analysis described above to reconstruct two networks for each cancer type: one using samples of that cancer and another using samples of all other 17 cancers. Then, we constructed a differential network by identifying edges that are present in the former network but not in the latter. To ensure the fairness of comparisons, we focused on the same subset of genes that were utilized by InPheRNo.
  We would like to note that DiNA is indeed a useful method in removing unwanted edges (e.g., those corresponding to a confounding effect), and we used it in this study to correct for the confounding effect of tissues of origin of each cancer type. However, when DiNA is used for the different problem of identifying phenotype-relevant TRNs, it misses on important edges such as those shown in Fig. 1b and 1c.
4. *Context-specific TRN reconstruction:* this is another class of methods in which genes associated with phenotype variation are identified, e.g., by differential expression analysis, and then a network is constructed by relating the expression of these genes to the expression of TFs^17–19^ (Supplementary Fig. 1c in Supplementary Information). As one of our baseline methods, we implemented this approach by first identifying top 1500 genes that were differentially expressed between one cancer type compared with other types of cancer (Bonferroni-corrected *p* < 1E-20). Then, we used Elastic Net to relate the expression of these genes to the expression of TFs and construct a TRN using the TFs with nonzero coefficients.
5. *Master regulator analysis (MRA):* MRA^20^ is a method for identification of key TFs whose targets are enriched for a set of phenotype-associated genes (e.g., differentially expressed genes). MRA does not construct a TRN, but rather accepts a TRN as input (along with a set of TFs and a set of phenotype-associated genes) and utilizes this network to rank TFs that may influence the phenotype. Although this method solves a problem different than the one addressed by InPheRNo, for completeness we included it in our analyses as a benchmark. We used the MRA-FET implementation of this approach in geWorkbench^39^ for the analysis. Similar to the context-specific approach above, we used top 1500 genes that were differentially expressed between one cancer type compared with other types of cancer (Bonferroni-corrected *p* < 1E-20) as the phenotype-associated genes, and used the TRN constructed using Elastic Net as the input network.

### Randomized degree-preserving network and degree distribution-preserving networks tests

To calculate the empirical *p* values for a randomized degree-preserving networks test, to evaluate the enrichment of InPheRNo networks in global networks identified using TREG and ChIP-seq data, we generated 5000 random networks using the code available in (http://maslov.bioengineering.illinois.edu/matlab.htm)^93^. To calculate the empirical *p* values based on a randomized degree distribution-preserving networks test, we generated 5000 random networks by randomly permuting the TF and gene labels.

### Survival analysis using gene expression profiles and gene signatures

The results reported in Fig. 4a correspond to nine different approaches in clustering of the samples of each cancer type into two groups: five correspond to clustering based on gene expression signatures, where the remaining four utilize the gene expression data itself. For the methods that utilize a gene expression signature, we defined the signature of a TF in each cancer type as a weighted linear combination (${\boldsymbol{x}} + \mathop {\sum}\nolimits_i {w_i{\boldsymbol{y}}_i}$) of the expression profile of the TF (denoted by ***x***) and its targets (denoted by ***y***_*i*_) across different samples of that cancer type. Note that using a weighted average of gene expression profiles is a commonly used approach for forming polygenic gene expression signatures (e.g., see ref. ^94^). Consistent with previous analysis above, we used the Pearson’s correlation coefficient between the expression profile of the TF and each target gene as the weights (*w*_*i*_s) in this linear combination, to reflect the strength and mode of regulation of each gene. This signature represents the expression of the TF as well as the activity level of its targets, whereas considering the mode and strength of regulation. For each TRN reconstruction method, we used the signatures of *n*_*s*_ = 5 expressed TFs with the most identified targets, to cluster samples into two distinct groups for survival analysis (see Supplementary Methods and Supplementary Table 2 for more information on sensitivity of the results to the chosen value for *n*_*s*_).

We used the gene expression itself in four different ways. First, for each cancer type, we identified top *n*_*s*_ = 5 differentially expressed genes (DEGs) and used those as features to cluster samples into two groups (denoted as “Expression (Top DE genes)” in Fig. 4a). We selected the *n*_*s*_ = 5 to match the analysis done using gene signatures. As the signatures formed by InPheRNo combines both top five TFs and their target genes, we argued that its good performance may be due to using a large number of genes for clustering. To address this concern, we selected *k* DEGs, were *k* was equal to the number of genes we used to form the corresponding InPheRNo signature, and clustered the samples based on the expression of these genes (denoted as “Expression (Matched DE genes)” in Fig. 4a). Third, we used top *n*_*s*_ = 5 differentially expressed TFs for clustering (denoted as “Expression (To DE TFs)” in Fig. 4a). Finally, we used top *n*_*s*_ = 5 master regulators (based on MRA analysis) and used their expression for clustering (denoted as “Expression (Master regulators)” in Fig. 4a).

In all cases, we used agglomerative clustering with average linkage and cosine similarity, since it has been shown to be one of the best options for clustering of cancer samples using gene expression data^95^. Note that we chose the number of clusters a priori as two, to avoid the difficulties associated with identifying the “best” number of clusters (e.g., contradictions based on different metrics), while providing a (coarse) grouping of the samples based on their expression profiles.

### Reporting summary

Further information on research design is available in the Nature Research Reporting Summary linked to this article.

## Supplementary information

Supplementary Information

Supplementary Data 1

Supplementary Data 2

Supplementary Data 3

Supplementary Data 4

Supplementary Data 5

Supplementary Data 6

Supplementary Data 7

Supplementary Data 8

Supplementary Data 9

Reporting Summary

## Data Availability

The adjacency matrices corresponding to cancer type-relevant TRNs (not tissue corrected) generated from TCGA are provided in Supplementary Data 1. The adjacency matrices corresponding to tissue type-relevant TRNs generated from GTEx are provided in Supplementary Data 2. The adjacency matrices corresponding to tissue-corrected cancer type-relevant TRNs are provided in Supplementary Data 4.

## Code availability

Implementations of InPheRNo and simplified-InPheRNo in python, with appropriate documentation, are available at: https://github.com/KnowEnG/InPheRNo and https://github.com/KnowEnG/Simplified-InPheRNo, respectively.
