# Supplementary material for: Inference of phenotype-relevant transcriptional regulatory networks elucidates cancer type-specific regulatory mechanisms in a pan-cancer study
Source: NPJ Syst Biol Appl. 2021 Feb 8;7:9. doi: 10.1038/s41540-021-00169-7 (PMC7870953; doi:10.1038/s41540-021-00169-7)
Supplement: Supplementary file 1 — Supplementary Information [file 41540_2021_169_MOESM1_ESM.pdf]

Supplementary Information for "Inference of phenotype-relevant transcriptional regulatory networks elucidates cancer type-specific regulatory mechanisms in a pan-cancer study"

Amin Emad[1] and Saurabh Sinha[2, 3, 4]

[1] Department of Electrical and Computer Engineering, McGill University, Canada

[2] Carl R. Woese Institute for Genomic Biology, University of Illinois at Urbana-Champaign, USA

[3] Department of Computer Science, University of Illinois at Urbana-Champaign, USA

[4] Cancer Center at Illinois, University of Illinois at Urbana-Champaign, USA

Corresponding Authors:

Amin Emad
755, McConnell Engineering Building
3480 University Street
Montreal, Quebec, Canada H3A 0E9
Email: amin.emad@mcgill.ca

Saurabh Sinha
2122 Siebel Center
201 N. Goodwin Ave
Urbana, IL, USA 61801
Email: sinhas@illinois.edu

SUPPLEMENTARY METHODS

Normalization of the transcriptomic data

For the pan-cancer (or pan-tissue) normalization, first genes that had an FPKM (or RPKM) value of at least 1 for more than 100 samples were selected as "expressed". The expression values of the expressed genes in each sample were quantile normalized to follow the average empirical distribution observed across samples. Then, the expression values of each gene (across different samples) were inverse quantile normalized to a standard normal distribution. The cancer (or tissue) -specific normalization were done similarly, with the difference that expressed genes were defined as those with FPKM (or RPKM) values of at least 1 for more than 10 samples of each cancer (or tissue) type. Note that for the analysis of GTEx data, only samples with RNA integrity number (RIN) of at least 6 were selected.

Hyperparameters of Elastic Net

We used Elastic Net in several TRN reconstruction approaches. For this purpose we used the function ElasticNetCV implemented in the scikit-learn library (version 0.18.1) for python [1]. This library implements Elastic Net by minimizing the objective function,

$$\frac{1}{2n}\|y - Xw\|_2^2 + \alpha\rho\|w\|_1 + \frac{1}{2}\alpha(1-\rho)\|w\|_2^2, \qquad (1)$$

where n is the number of samples, y is the response vector, X is the feature matrix, w is the unknown vector of coefficients, and α and ρ are hyperparameters. The hyperparameter α is chosen using cross-validation by iteratively fitting the model along a regularization path. We used the default value of the library for ρ (ρ=0.5).

Parameters of InPheRNo used to reconstruct cancer type-relevant TRNs

The hyperparameters of the Elastic Net used in the first step of InPheRNo were selected as described above with the additional constraint that the maximum number of nonzero coefficients in the learnt model should be at most equal to $m_{\max} = 15$, to reduce the computational complexity and impose the prior knowledge that only a few TFs regulate each gene. Note that imposing an upper limit on the number of regulators of a

gene has been previously used for various reasons including the reasons above [2-4]. The value of γ (the prior probability of $T_{i,j} = 1$) was set as $\gamma = \frac{1}{10\ m_{\max}}$. The value of α' was estimated by fitting a mixture of a uniform and a $Beta(\alpha = \alpha', \beta = 1)$ distribution to the gene-phenotype association p-values of all the genes. All other unknown parameters were inferred automatically during the training of the PGM on the data.

We used a Markov chain Monte Carlo (MCMC) method using the PyMC python module (version 2.3.6) [5] to infer the unknown parameters and learn posterior probabilities for $T_{i,j}$s. The MCMC was initialized randomly and 200 samples were obtained for each latent variable. The last 100 samples were used to obtain an empirical posterior probability for $T_{i,j} = 1$, for each (i, j) pair. Since some of the solutions of the MCMC may converge to local optima, to alleviate their effect we ran the MCMC procedure 100 times with different random initializations and obtained an average posterior probability for each $T_{i,j}$. These average values were then minmax normalized and a threshold of 0.5 was used to identify phenotype-relevant regulatory edges.

For each cancer type, the networks were constructed for the union of the top 1,500 most differentially expressed genes for that cancer (versus other cancer types) and the top 1,500 most differentially expressed genes for the corresponding normal tissue (versus other normal tissues).

Accuracy and robustness analysis of InPheRNo

InPheRNo utilizes a multivariable regularized regression method (Elastic Net) to relate the expression of a gene to the expression of candidate TFs and short-list a small set of TFs to be used in the PGM. In order to evaluate the accuracy of this step, we used a cross-validation framework. For this purpose, we focused on genes and TFs utilized to construct the COAD-relevant TRN using InPheRNo. We randomly divided the set of samples into two groups: one for training and one for evaluating the accuracy (the test set). Then, for each gene, Elastic Net (with the same parameters as was used in InPheRNo) was utilized to learn a model relating

the expression of the gene to the expression of a small set of TFs for the training set. Then this trained model was used to predict the expression of each gene for the samples in the test set. These analyses revealed that the predicted gene expression values have a high concordance with the true expression values in the test set (median Spearman's rank correlation = 0.72, p < 1E-16).

Next, we sought to determine the sensitivity of InPheRNo to the number of samples. For this purpose, we randomly selected 36 samples from each cancer type (648 samples in total), equivalent to approximately a 90% reduction in the number of samples, making the number of samples smaller than the total number of TFs (i.e., features). Then, we reconstructed the COAD-relevant TRN using the same parameters used to construct the original network. Comparing the edges identified by all the samples and by ~10% of the samples revealed that more than 67.2% of the recovered edges are shared between the two networks (p < 1E-16, hypergeometric test). We believe this high degree of consistency, in spite of this drastic reduction in the number of samples, is due to utilizing a 'regularized' regression model (Elastic Net) followed by a systematic integration of phenotypic and transcriptomic information using a PGM.

Reconstruction of breast cancer PAM50 subtype-relevant TRN

We obtained the PAM50 molecular subtype annotations of 839 TCGA BRCA samples from UCSC Xena [6]. For this purpose, we used the "PAM50Call_RNAseq" column of the file available at the address https://tcga.xenahubs.net/download/TCGA.BRCA.sampleMap/BRCA_clinicalMatrix.gz. In addition, we obtained the gene expression profile (HTseq counts) of the same patients from the Genomic Data Commons [7]. We used the package EdgeR [8] and fit a negative binomial generalized log-linear model to the read counts for each gene, while correcting for the confounding effects of sex and race, to a p-value of association between the gene expression and PAM50 subtypes for each gene. For the follow-up analysis, we focused on 460 genes with the smallest p-value (Bonferroni-corrected p^{*}<1E-159). Separately, we performed a quantile normalization using voom [9] and a z-score transformation (across the samples) on the gene expression profiles

and used the transformed values to obtain the pseudo p-values of gene-TF associations. All parameters of InPheRNo were identical to those used for reconstructing cancer type-relevant TRNs.

Supplementary Data 6 provides the final TF-gene scores assigned by InPheRNo (aggregated over 100 runs) and Supplementary Data 7 provides the list of TFs with the greatest number of PAM50 subtype-relevant targets in this subnetwork and literature evidence for their role in different subtypes of BRCA. In this table, 14 TFs were identified as regulators of at least 1% of the considered genes. Five of these TFs were also implicated by InPheRNo in BRCA-relevant TRN and were among TFs with the greatest number of target genes in Table 2. In addition, among these TFs, FOXA1, MYB, and GATA3 are drivers of BRCA according to both IntOGen [10] and DriverDBv2 [11]. In what follows, we provide a summary of the literature evidence in support of the role of these TFs in different subtypes of breast cancer.

The role of FOXC1, a member of the forkehead family of TFs, in the development, progress, and metastasis of BRCA and particularly basal-like subtype is well established, and this TF is considered both a diagnostic and a prognostic biomarker of basal-like subtype of breast cancer [12-15]. The role of FOXA1, another member of the forkhead family, in breast cancer and particularly the luminal subtype is well documented [16-18]. In addition, we recently showed that FOXA1 and FOXC1 act as biomarkers of metastatic subtypes of breast cancer [19]. FOXC2, has been shown to play important roles in breast cancer metastasis, and its expression has been shown to be correlated with aggressive basal-like subtype of breast cancer [20,21]. FOXM1, another member of the forkhead family of TFs, has been suggested as a prognostic factor and potential therapeutic target in breast cancer [22] and particularly the triple-negative subtype [23]. SOX8, a member of SOX family of TFs that are involved in embryonic development, is expressed in triple-negative breast cancer [24] and has been recently suggested to be involved in the development and progress of this subtype [25]. TFCP2L1, which plays a crucial role in maintenance of embryonic stem cells, has also been implicated in breast cancer [26]. ESR1 encodes estrogen receptor alpha and has been shown to play a crucial role in the development, progress and drug

resistance of breast cancer [27-29]; in addition, the expression of this TF has been shown to be predictive of molecular subtypes of breast cancer [30,31]. MYB (c-Myb) and MYBL2 (b-Myb), are two members of the MYB family of TFs and have been implicated in various aspects of breast cancer including, including cell proliferation, cell apoptosis, drug resistance, patient survival, and metastasis [32-37]. E2F3 has also been suggested as a diagnostic biomarker and potential therapeutic target of breast cancer [38] and its silencing has been shown to suppress the tumor growth of Her2+ breast cancer cells [39]. GATA3 is a driver of breast cancer and its various role in breast cancer development, progress, and metastasis has been well-documented [40-42]. Finally, BCL11A has been implicated in aggressive triple-negative breast cancer and has been suggested as a potential therapeutic target for this subtype [43-45].

The evidence above highlights the power of InPheRNo in revealing gene expression programs related to a phenotype (here the PAM50 subtypes of breast cancer), and its ability to identify key regulators, diagnostic and prognostic biomarkers, and therapeutic targets by combining phenotypic information with gene and TF expression.

Sensitivity of the survival analysis results to the number of TFs considered in the gene signatures

To obtain the results depicted in Figure 4, we used $m_s = 5$ TFs with the greatest number of targets to form the gene signatures based on different TRN reconstruction methods (including InPheRNo) and cluster patients of different cancer types into two groups. Similarly, $m_s = 5$ most differentially expressed TFs and genes were also used as alternative features for clustering and follow-up survival analysis. To test the sensitivity of the results to this number, we repeated the survival analysis with different values of $m_s = 5, 15, 25, 35, 45, 55, 65, 75$. These values were used for signatures formed using all methods (including InPheRNo) as well as methods that used gene expressions directly in the exact same manner as in Figure 4a (a total of 9 methods). Supplementary Table 2 shows the number of cancer types (out of 13) for which InPheRNo signature resulted in two groups with distinct survival ($p < 0.05$, logrank test), as well as its rank

compared to other methods in having the largest number of cancers with distinct survival (1 being the best an 9 being the worst). As can be seen in this table, except for $m_s = 45$ in which InPheRNo signature results in the second-best performance, in all other cases, the signature formed by InPheRNo is consistently outperforms all other eight methods.

Simulation analysis for the distribution of pseudo p-values of gene-TF associations

As we discussed in the Methods section, we expect that the pseudo p-values of gene-TF associations obtained using the two-step procedure of Elastic Net followed by ordinary least square (OLS) regression would not follow a uniform distribution even when a TF does not regulate a gene, instead exhibiting a distribution biased towards small values. To test this, we first randomly generated expression profiles of $n_{TF} = 100$ TFs. The profile of each TF is a vector of length $n_{\text{sample}} \in \{80, 100, 120\}$, representing the expression of that TF in n_{sample} samples. These choices of n_{sample} correspond to cases where the number of samples is smaller, equal and larger than the number of features (i.e., TFs). The expression of each TF in each sample follows an independent identically distributed standard Normal distribution (mean = 0 and variance = 1). To generate the expression of a gene $\boldsymbol{y}$, we assumed a linear relationship between its expression and the expression of its regulators according to

$$\boldsymbol{y} = \sum_{i=1}^{k} w_i \boldsymbol{x}_i, \quad (2)$$

where $\boldsymbol{x}_i$ is the expression profile of TF i, w_i represents strength and mode of regulation and follows a Uniform(-1, 1) distribution, and k is the number of regulators of the gene. To form this profile we selected k TFs (out of 100) randomly. We will discuss different choices of k in what follows. Next, we used z-score normalization to normalize the expression of each gene. To consider both noise-free and noisy data, we added random Gaussian noise with mean equal to 0 and standard deviation equal to 5% of the standard deviation of the gene's expression profile.

We used the two-step procedure described in Methods to obtain the pseudo p-value for each gene-TF pair. In the first step of the procedure, we used a multivariable Elastic Net with the parameters selected as described earlier. These parameters were the same as those we used to analyze the pan-cancer dataset using InPheRNo, including the maximum number of candidate TFs $m_{\max} = 15$. Since the true number of regulators of a gene are not known in advance, and may be smaller than, equal to or larger than the value we chose for $m_{\max}$, we considered three options of k = 13, 15, 17 corresponding to these scenarios, respectively. We repeated this procedure 1000 times and obtained empirical distributions of pseudo p-values for TF-gene associations when the TF regulates the gene and when the TF does not regulate the gene. The mean, standard deviation, median and skewness of the empirical distributions for different models are provided in Supplementary Table 4 and the histograms of these distributions are shown in Supplementary Figures 10-12. (Due to the significant bias of the pseudo p-values towards small values when the TF regulates the gene, in addition to linear plots we have used semilog plots as well to better show these small values). Note that skewness [46] is a measure of asymmetry in a distribution; a positive skewness shows that most of the mass of the distribution is concentrated on the left side (e.g. values close to zero in our case), a negative value shows that most of the mass is concentrated in the right side and a value of zero shows that the distribution is symmetric (e.g. a uniform distribution).

It is clear from these results that the distribution of pseudo p-values is biased towards smaller values even when the TF does not regulate the gene (a positive value of skewness). In addition, for each model, comparing the distribution of pseudo p-values when a TF does not regulate a gene with the case in which a TF regulates a gene, shows that the bias towards smaller values is much more significant in the latter (for example compare skewness of 0.43 and median of 0.33 to skewness of 38.1 and median of 2.0E-59 for 80 samples and $k = 13$ in the noiseless model).

Reasons for using the two-step procedure for obtaining pseudo p-values of gene-TF associations

Currently, developing methods for obtaining p-values of regression coefficients for regularized regression models such as LASSO and ElasticNet is an active area of research in statistics. Several approaches have been developed based on resampling or data splitting [47,48]. However, since in typical TRN reconstruction problems, the number of samples (or conditions) is usually much smaller than the number of features, resampling or splitting the data results in erroneous estimates of p-values. Since our goal was to develop a method that is applicable to a wide range of problems, many of which suffer from small samples sizes, we decided not to use this method. Recently, Lockhart, et al. [49] suggested a new approach for estimating the p-value of feature-response association in a regularized model (LASSO). While this work is indeed a break-through in this area, its proposed statistical test does not calculate the p-values necessary for our setup, and at best is an alternative way of calculating pseudo p-values (see details below). In addition, in the setup considered in [49] (which differs from the setup necessary in our approach), the authors consider several assumptions necessary for their proofs, which do not hold in our more general case. Given the above challenges, we decided to use OLS, due to the efficient methods available for calculating p-values, and the fact that based on our simulations we could estimate the p-value distributions under the Null hypothesis using a Beta distribution. In what follows, we provide the details of why the setup considered in [49] differs from our case.

The statistical test: As the authors mention in the Abstract [49], they consider "testing the significance of the predictor variables that enters the current lasso model, in the sequence of models visited along the lasso solution path". This is different from the setup necessary for our case, in which a lasso/ElasticNet model is trained on the data for some value of λ (the L1 regularization parameter) and we would need to calculate a p-value for each of the selected predictors. In fact, the authors clearly state this in their paper: "The problem of assessing significance in an adaptive linear model fit by the lasso is a difficult one, and what we have presented in this paper by no means a complete solution", followed by their plans for future work to address the general case (the case relevant to our setup): "Significance test for generic lasso models. A natural

direction to consider is the generic lasso testing problem: given a lasso model computed at some fixed value of λ, how do we carry out a significance test for each predictor in the active set? Work on this is in progress."

The problem setup they consider is defined on page 417 as "at a given step in the lasso path (i.e., at a given knot), we consider testing the significance of the variable that enters the active set." In other words, the test suggested in this paper is an "adaptive" one that depends on both the lasso path as well as the already selected predictors in the set in the kth step. Both of these dependencies are important and make the setup distinct from that necessary for our problem. As authors have pointed out on page 416, "when rank(X)<p there are many lasso solutions at each λ and, therefore, possibly many solution paths". As a result, this dependency requires an assumption on the columns of X (i.e. "the columns of X are in general position"), which may not be satisfied in the general case (see footnote 7 on page 417 for precise definition of the "general position").

The second dependency (i.e. the dependency on the already selected predictors) is a much more important issue. In our setup, the reason we seek p-values is to have a measure of relevance between the predictors and the response, and therefore the order that the predictors (i.e. TFs) are selected for each gene should not affect the p-value. However, the test suggested in this paper does not satisfy this condition. As the authors mention in Remark 2 (page 420) "Our test is specific to a step in the path, and not to a predictor variable at large". Moreover, in Remark 4 (same page), they clearly state that "By design, the covariance test is applied in a sequential manner, estimating p-values for each predictor variable as it enters the model along the lasso path. A more difficult problem is to test the significance of any of the active predictors in a model fit by the lasso, at some arbitrary value of the tuning parameter λ", and they refer the readers for this more general (and relevant to our setup) case to future work (see page 453).

The Null distribution: The null hypothesis in this test differs from that of a general lasso (which is of the interest to our study). More specifically, as stated on page 424, "Given a fixed number of steps k ≥ 1 along the lasso path, the covariance test examines the set of variables A selected by the lasso before the kth step (i.e., A is the current active set not including the variable to be added at the kth step)." The difference in the Null distribution, once again points to the two dependencies described above: the dependency on the specific lasso path and the dependency on A (i.e. the already selected predictors).

The Assumptions: Several assumptions are considered for the statistical test, which may not be satisfied in our practical setup. These include the columns of X being in general position (page 417), as well as various stricter assumptions for the non-orthogonal X case in section 4 such as equations (26) and (27).

Given the major difference above and the fact that the statistical test developed in this paper does not work in the general setup of LASSO or EN, we decided not to use this method for our analysis, since it can only provide pseudo p-values for our case of interest, and once again we need to model their distribution under the Null hypothesis with a non-uniform distribution, similar to what we did using OLS. On the other hand, since OLS has other benefits such as an efficient method of calculating p-values, and a well-approximated distribution in our problem (see the simulations discussed earlier), we concluded that OLS is a more appropriate choice for obtaining the p-values for our application.

False positive analysis of TF-gene associations

Association between expression of genes and TFs are widely used to identify TRNs and various methods exist to perform this task (e.g. ARACNE [50], ASTRIX [3], etc.). To show that InPheRNo does not generate a large number of false positives by using summary statistics instead of all the data, we compared the distribution of p-values

of Pearson correlation between the expression of *all* gene-TF pairs in the TCGA dataset with the distribution of the gene-TF pairs *reported by InPheRNo* for each cancer type.

First, we used the skewness as the measure of how much the distributions are biased towards small values: a larger positive skewness value shows that most of the weight of the distribution is gathered in the left side (i.e. small p-values). The skewness of all gene-TF p-values was equal to 4.1, while the skewness of the gene pairs identified as edges by InPheRNo ranged from 126.4 to 171.7 (Supplementary Table 5), showing a very large bias towards small p-values. In addition, we compared the enrichment of the set of TF-gene pairs identified by InPheRNo (as caner type-relevant TRN edges) with TF-gene pairs whose correlation p-value was among the smallest 2% and 10% of all the p-values. Supplementary Table 5 shows that more than 50% of InPheRNo edges were among the smallest 2% and more than 89% were among the smallest 10% p-values of all pairs (enrichment p-values were extremely significant in all cases). These results show that while InPheRNo does not simply identify the smallest TF-gene p-values, the edges identified by it are biased towards such small p-values. Consequently, utilizing p-values as input to the PGM of InPheRNo does not generate a large number of false positives: InPheRNo-identified edges correspond to TF-gene pairs whose expression correlation p-values are biased towards small values.

SUPPLEMENTARY DATA

Supplementary Data 1: The file contains the adjacency matrices of cancer type-relevant TRNs (not tissue-corrected) obtained using InPheRNo and TCGA data.

Supplementary Data 2: The file contains the adjacency matrices of tissue-relevant TRNs obtained using InPheRNo and GTEx data.

Supplementary Data 3: The p-values of overlap between InPheRNo-identified TRNs for different cancers (and tissues) and global TRNs identified by TREG using ChIP-seq data. This table contains results of the randomized degree-preserving networks test and the randomized degree distribution-preserving networks test.

Supplementary Data 4: The file contains the adjacency matrices corresponding to tissue-corrected cancer type-relevant TRNs obtained using InPheRNo.

Supplementary Data 5: List of top 100 TFs identified using different methods. Each sheet corresponds to a different cancer type and each column in a sheet contains a ranked list of 100 TFs identified using the method specified in the column header.

Supplementary Data 6: The final TF-gene scores obtained using InPheRNo (aggregated over 100 runs) for BRCA PAM50 subtype-relevant TRN.

Supplementary Data 7: The ranked list of TFs with the greatest number of PAM50 subtype-relevant targets in the PAM50 subtype-relevant TRN constructed by InPheRNo.

Supplementary Data 8: The gene ontology (GO) enrichment of top 5 TFs and their targets identified using InPheRNo for LGG. The odd numbered sheets contain the enriched GO terms and their enrichment p-values for each TF and its targets, while the even numbered sheets contain the list of targets for each TF identified using InPheRNo. The enrichment analysis was performed using the KnowEnG platform (knoweng.org) and raw p-values (Fisher's exact test) as well as p-values adjusted for multiple hypothesis testing are reported.

Supplementary Data 9: The gene ontology (GO) enrichment of top 5 TFs and their targets identified using InPheRNo for PAAD. The enrichment analysis was performed using the KnowEnG platform (knoweng.org) and raw p-values (Fisher's exact test) as well as p-values adjusted for multiple hypothesis testing are reported.

SUPPLEMENTARY TABLES

Supplementary Table 1: Number of samples for each tissue type obtained from GTEx.

Name of the tissue	Number of samples
Adrenal Gland	140
Brain	1171
Breast	188
Colon	298
Esophagus	647
Liver	104
Lung	298
Ovary	92
Pancreas	162
Prostate	88
Skin	575
Stomach	180
Testis	153
Thyroid	292

Supplementary Table 2: The effect of m_s (number of TFs or genes), used for clustering, on the results of survival analysis reported in Figure 4a. The second row shows the number of cancer types (out of 13) in which clusters identified based on InPheRNo signature showed distinct survival probabilities in Kaplan-Meier analysis ($p < 0.05$, log rank test). The third row shows the standing of InPheRNo signature compared to eight other methods: 1 means the method resulted in the largest number of cancer types with significant p-values.

$\boldsymbol{m_s}$	5	15	25	35	45	55	65	75
**InPheRNo's Num. Significant cancer types (out of 13)**	7	6	6	7	5	5	5	5
**Rank of InPheRNo (out of 9 methods)**	1	1	1	1	2	1	1	1

Supplementary Table 3: The p-values (log rank test) of the survival analysis performed for each cancer type using signatures formed based on different approaches. Each row corresponds to a different cancer type and each column corresponds to a different approach.

	InPheRNo (signature)	Simplified-InPheRNo (signature)	Context-restricted (signature)	DiNA (signature)	Context-specific (signature)	Most differentially expressed genes	Most differentially expressed genes (number matched with InPheRNo signature)	Most differentially expressed TFs	MRA
**BRCA**	0.70	0.69	0.22	0.71	0.41	0.074	0.54	0.96	0.14
**COAD**	2.3E-2	0.19	0.80	0.089	0.61	0.77	4.4E-2	0.25	0.38
**ESCA**	0.93	0.54	0.64	0.56	0.30	0.82	0.79	0.93	0.52
**GBM**	4.7E-2	0.95	0.10	0.13	4.2E-2	0.84	0.94	0.053	0.65
**LGG**	3.1E-9	0.43	9.4E-6	8.5E-7	5.9E-10	1.0E-6	6.6E-6	2.6E-5	1.1E-4
**LIHC**	4.9E-2	1.2E-2	0.63	0.069	0.055	3.0E-2	4.4E-4	0.76	0.13
**LUAD**	0.13	0.34	0.66	3.3E-2	0.054	4.6E-5	4.7E-2	0.27	0.50
**LUSC**	2.8E-2	2.4E-2	0.11	0.78	1.7E-2	0.054	0.066	2.0E-2	0.39
**OV**	0.89	0.09	3.7E-2	0.53	0.15	3.5E-3	0.19	0.96	0.31
**PAAD**	3.1E-3	0.19	0.16	0.055	2.4E-2	0.83	0.072	0.83	4.2E-2
**READ**	0.55	0.48	0.21	0.19	0.85	0.39	0.26	0.27	0.56
**STAD**	0.15	2.5E-2	0.24	0.25	0.75	0.49	0.22	0.074	4.9E-2
**THCA**	3.2E-2	0.20	0.15	0.82	0.068	0.54	0.66	0.92	0.28

Supplementary Table 4: Statistics of the empirical distributions of the pseudo p-values of gene-TF associations obtained by applying the two-stage procedure to the simulated data.

			Identified TF does not regulate gene				Identified TF regulates gene			
			**Mean**	**Std**	**Median**	**Skewness**	**Mean**	**Std**	**Median**	**Skewness**
**Num. Samples = 80**	**Num. TFs in model = 13**	**Noiseless**	0.39	0.30	0.33	0.43	6.1E-4	1.7E-2	2.0E-59	38.1
		**Noisy**	0.37	0.30	0.30	0.51	1.0E-3	2.1E-2	1.5E-38	29.4
	**Num. TFs in model = 15**	**Noiseless**	0.37	0.30	0.29	0.54	1.5E-3	2.8E-2	1.6E-27	25.4
		**Noisy**	0.35	0.29	0.26	0.66	1.8E-3	2.9E-2	1.6E-24	23.1
	**Num. TFs in model = 17**	**Noiseless**	0.32	0.29	0.22	0.76	2.5E-3	3.0E-2	5.0E-16	19.3
		**Noisy**	0.31	0.28	0.22	0.83	2.6E-3	3.3E-2	2.3E-15	20.4
**Num. Samples = 100**	**Num. TFs in model = 13**	**Noiseless**	0.37	0.31	0.31	0.44	1.2E-4	7.0E-3	2.2E-222	74.2
		**Noisy**	0.36	0.30	0.28	0.59	3.8E-4	1.2E-2	8.3E-62	55.8
	**Num. TFs in model = 15**	**Noiseless**	0.37	0.30	0.29	0.52	3.5E-4	1.3E-2	8.2E-56	48.1
		**Noisy**	0.36	0.30	0.28	0.54	5.9E-4	1.8E-2	2.5E-42	42.9
	**Num. TFs in model = 17**	**Noiseless**	0.34	0.29	0.27	0.63	9.4E-4	2.0E-2	1.9E-28	30.9
		**Noisy**	0.33	0.29	0.25	0.73	1.2E-3	2.3E-2	2.8E-25	29.3
**Num. Samples = 120**	**Num. TFs in model = 13**	**Noiseless**	0.32	0.31	0.23	0.64	1.5E-5	1.7E-3	<1E-300	112.3
		**Noisy**	0.31	0.28	0.22	0.83	1.8E-4	6.4E-3	4.1E-80	56.9
	**Num. TFs in model = 15**	**Noiseless**	0.40	0.30	0.35	0.39	3.6E-5	1.8E-3	3.3E-96	72.2
		**Noisy**	0.35	0.30	0.27	0.59	2.4E-4	7.4E-3	1.1E-59	44.1
	**Num. TFs in model = 17**	**Noiseless**	0.35	0.29	0.28	0.57	3.5E-4	1.1E-2	2.0E-44	42.9
		**Noisy**	0.33	0.29	0.23	0.71	4.3E-4	1.3E-2	4.3E-39	47.6

Supplementary Table 5: The enrichment of the set of TF-gene pairs identified by InPheRNo (as caner type-relevant TRN edges) with TF-gene pairs that had the 2% smallest and 10% smallest correlation p-values (among all pairs). The first column shows the cancer type. The second column shows the skewness of the distribution of p-values of Pearson correlation between expression of TF-gene pairs that were identified by InPheRNo as TRN edges. The third and fourth columns show the percent of InPheRNo-identified TF-gene pairs whose correlation p-values were among the smallest 2% and 10% of all p-values, respectively.

Cancer	Skewness	Percent in smallest 2%	Percent in smallest 10%
**ACC**	164.3	53.4	90.9
**PCPG**	168.4	60.0	92.8
**GBM**	165.8	67.9	94.5
**LGG**	170.2	72.3	96.4
**BRCA**	150.8	55.1	92.9
**COAD**	171.7	61.8	94.0
**READ**	126.0	60.7	93.7
**ESCA**	147.8	60.4	92.8
**LIHC**	159.7	53.0	93.1
**LUAD**	159.4	55.5	91.4
**LUSC**	168.1	58.7	93.5
**OV**	159.4	48.9	89.9
**PAAD**	159.4	51.9	90.5
**PRAD**	167.3	54.7	92.8
**SKCM**	168.6	53.0	90.4
**STAD**	165.7	60.1	92.9
**TGCT**	170.7	50.1	89.0
**THCA**	166.4	55.4	93.2

SUPPLEMENTARY FIGURES

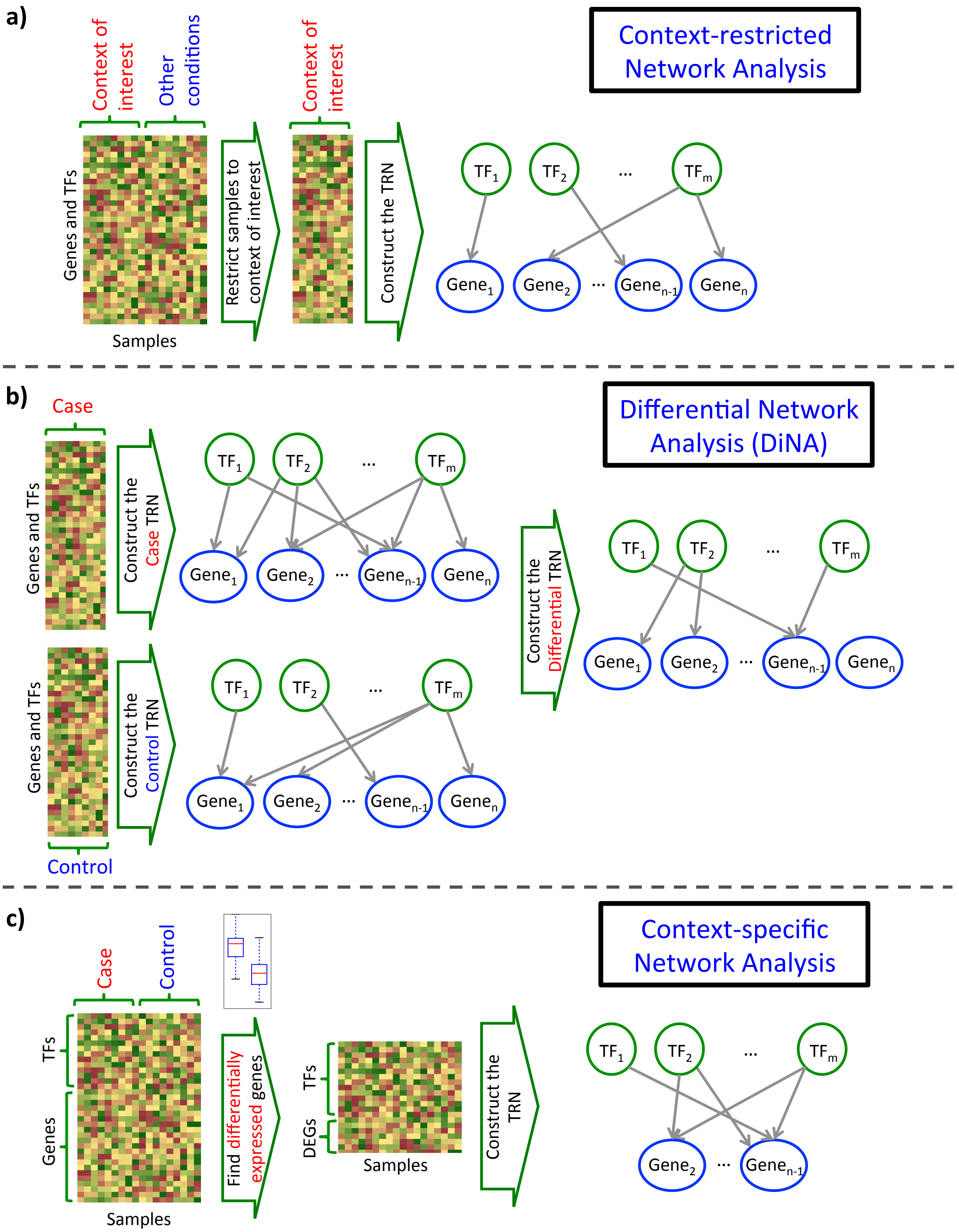

Supplementary Figure 1: The schematic of a) context-restricted, b) Differential network analysis (DiNA) and c) context-specific network analysis.

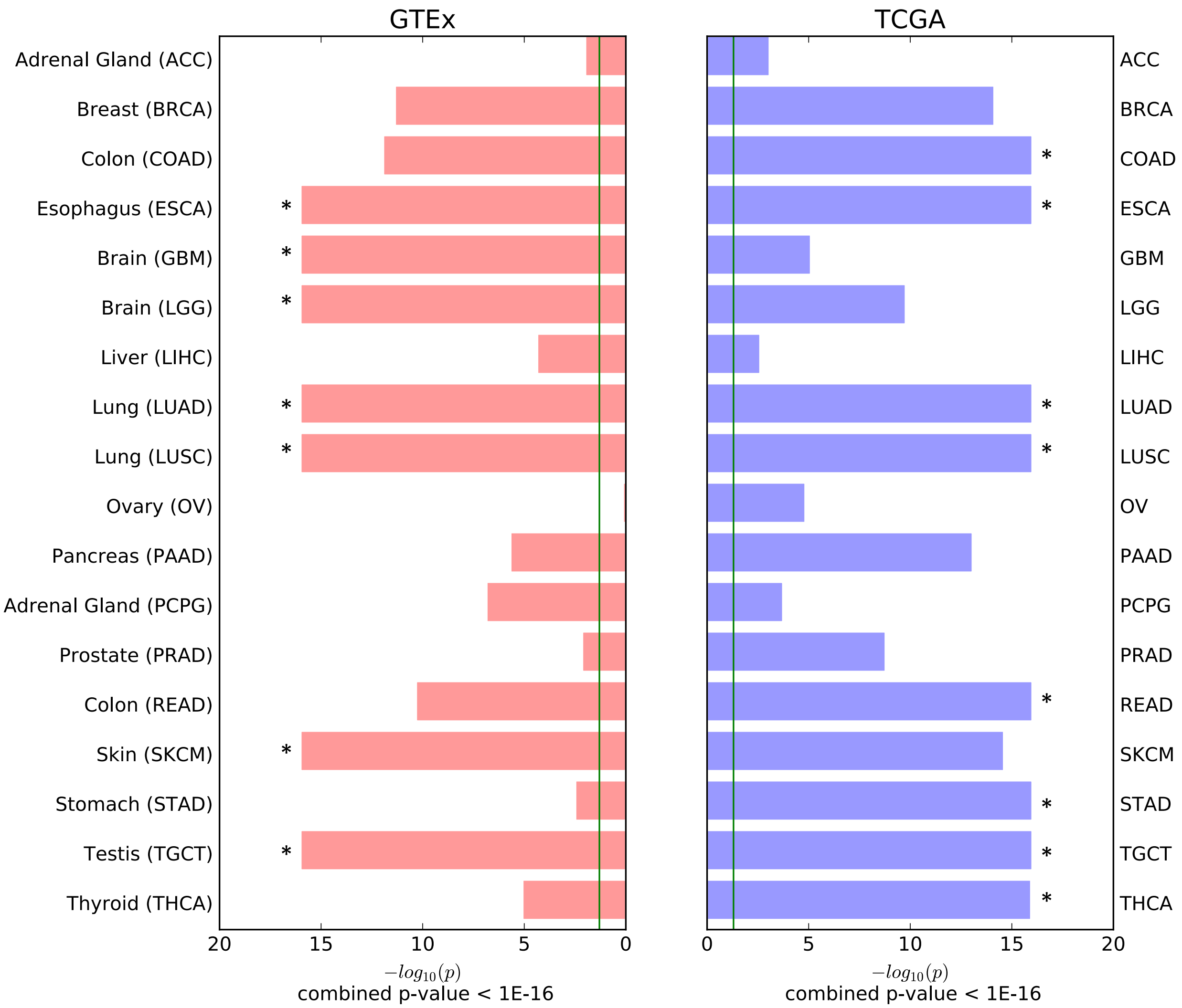

Supplementary Figure 2: The enrichment of InPheRNo-identified TRNs for different cancers (TCGA) and tissues (GTEx) in global TRNs identified by TREG. The bars represent $-\log_{10}(p)$ of enrichment (hypergeometric test). The green line shows the threshold alpha = 0.05 and the symbol * is used for cases in which $p < 1E-16$.

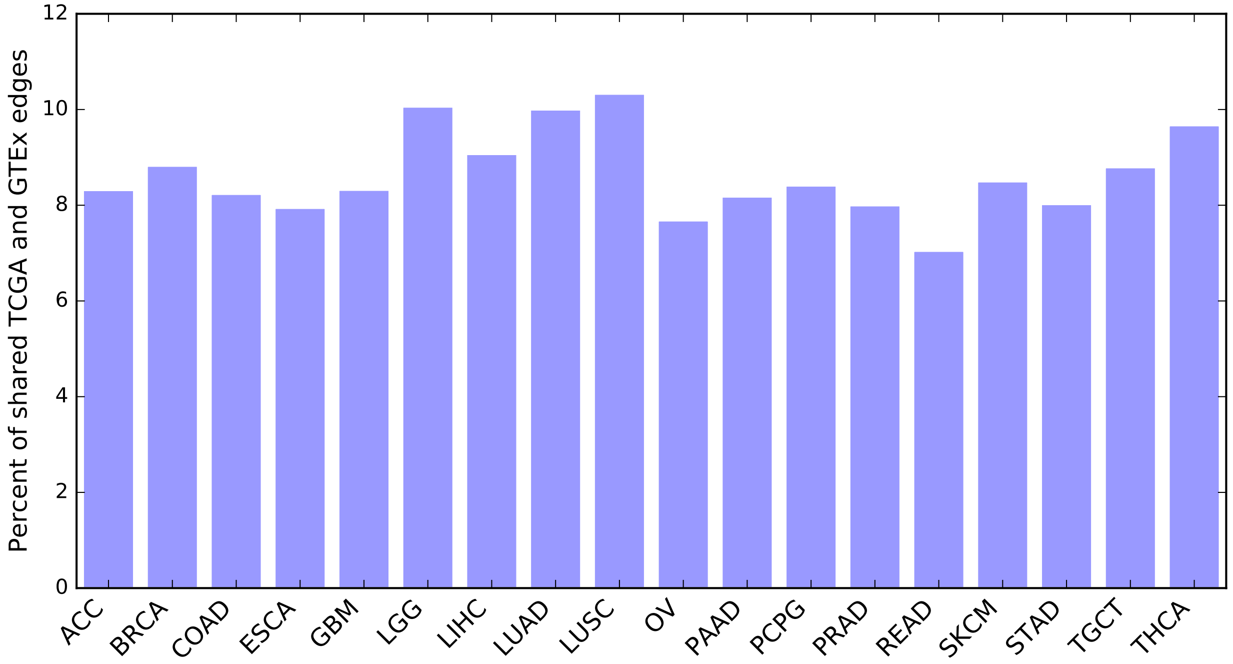

Supplementary Figure 3: The percent of shared edges in TRNs reconstructed using InPheRNo for each cancer type and their corresponding tissue. These shared edges were removed to correct for the confounding effect of tissues of origin.

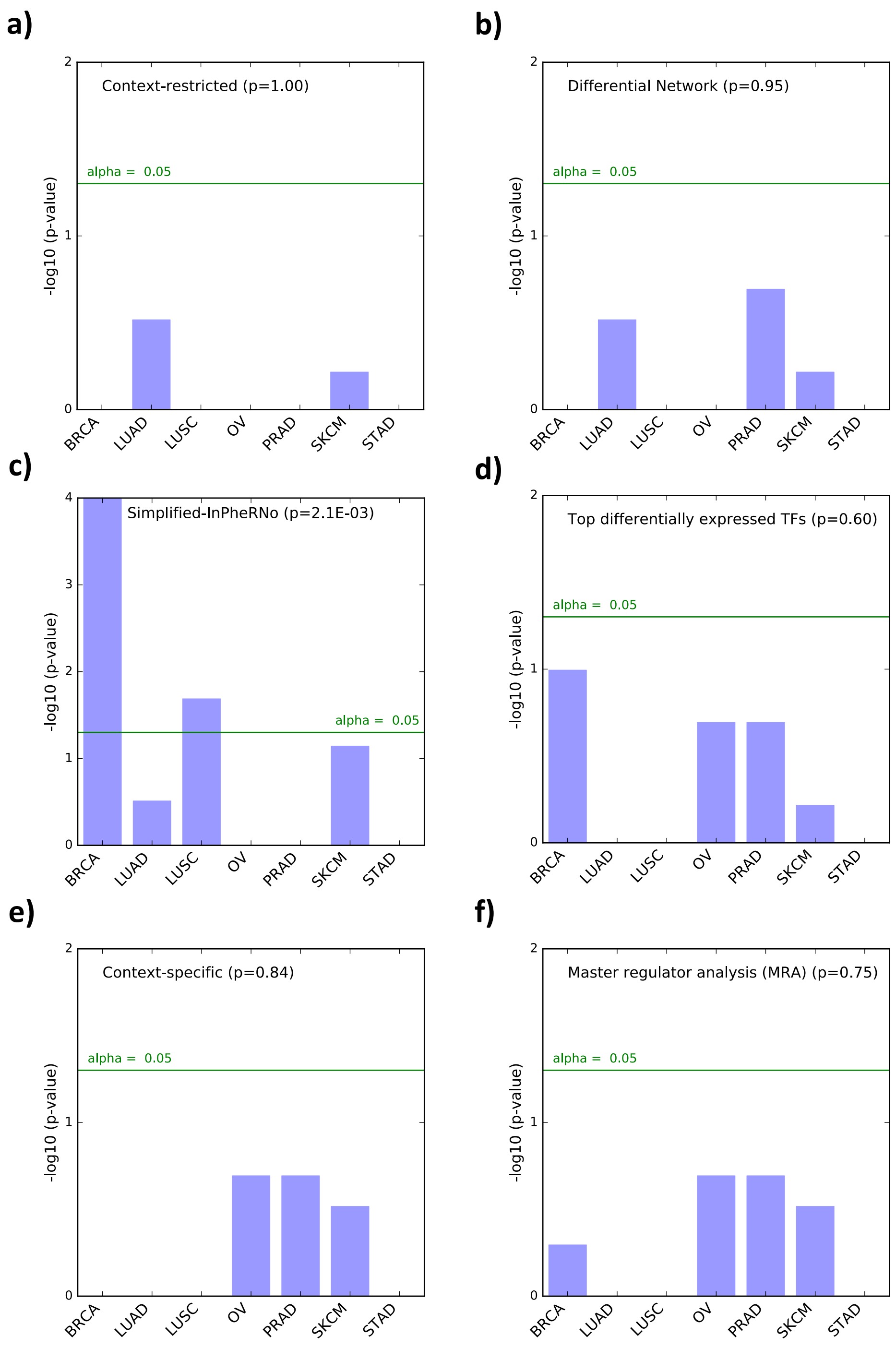

Supplementary Figure 4: Cancer-specificity of driver TFs identified using different methods. The bars represent the –log10 (p) of enrichment (hypergeometric test) of 100 TFs identified using different methods with driver TFs of that cancer type (annotated in the IntOGen database) that are drivers of at most $n_s = 2$ other cancers. The p-value shown in parenthesis represents the Meta p-value calculated using Fisher's method. (a) Results correspond to 100 TFs with most identified targets reconstructed using context-restricted network analysis. (b) Results correspond to 100 TFs with most identified targets reconstructed using differential network analysis (DiNA). (c) Results correspond to 100 TFs with most identified targets reconstructed by simplified-InPheRNo. (d) Results correspond to 100 TFs that were most differentially expressed in that cancer type compared to other cancers. (e) Results correspond to 100 TFs with most identified targets reconstructed using context-specific network analysis. (f) Results correspond to 100 key TFs identified using master regulator analysis (MRA).

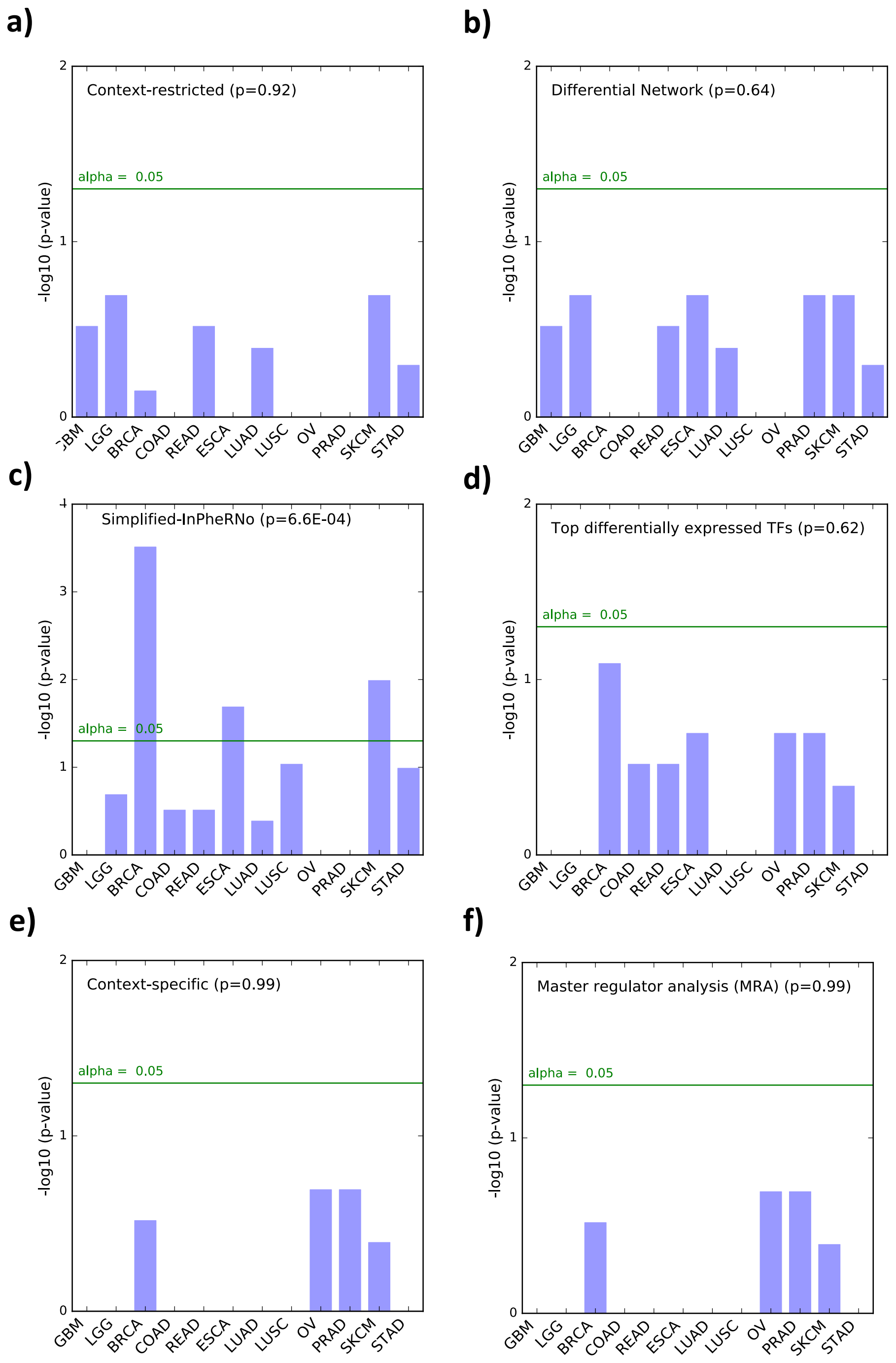

Supplementary Figure 5: Cancer-specificity of driver TFs identified using different methods. The bars represent the –log10 (p) of enrichment (hypergeometric test) of 100 TFs identified using different methods with driver TFs of that cancer type (annotated in the IntOGen database) that are drivers of at most $n_s = 3$ other cancers. The p-value shown in parenthesis represents the Meta p-value calculated using Fisher's method. (a) Results correspond to 100 TFs with most identified targets reconstructed using context-restricted network analysis. (b) Results correspond to 100 TFs with most identified targets reconstructed using differential network analysis. (c) Results correspond to 100 TFs with most identified targets reconstructed by simplified-InPheRNo. (d) Results correspond to 100 TFs that were most differentially expressed in that cancer type compared to other cancers. (e) Results correspond to 100 TFs with most identified targets reconstructed using context-specific network analysis. (f) Results correspond to 100 key TFs identified using master regulator analysis (MRA).

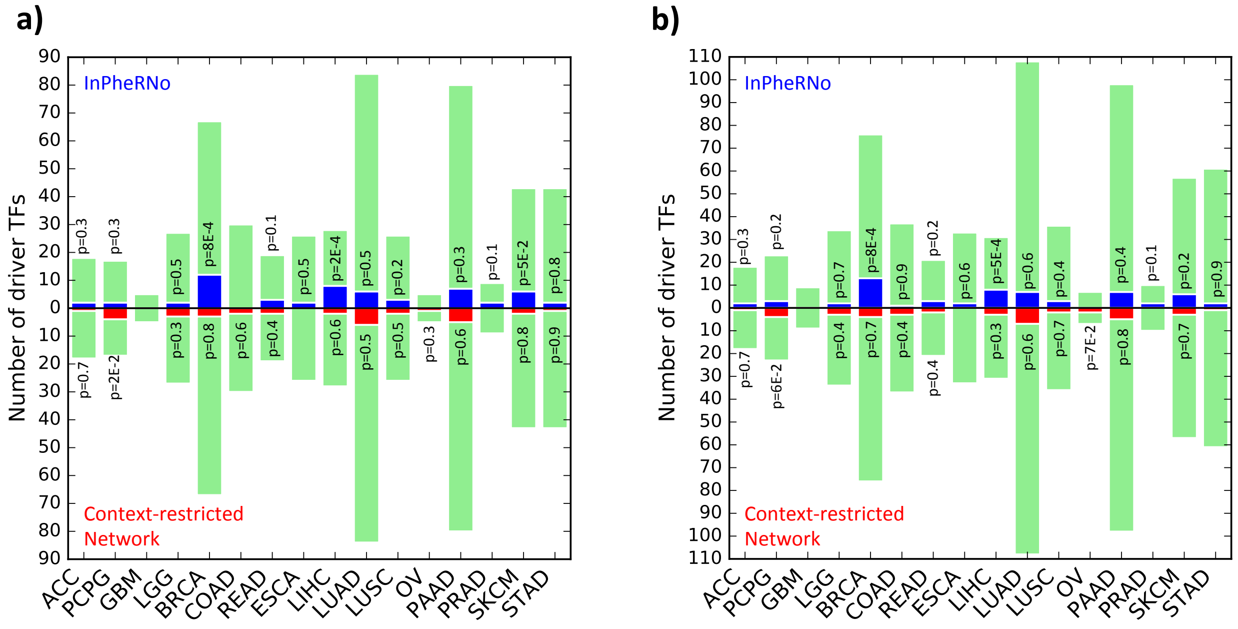

Supplementary Figure 6: Number of identified cancer-specific driver TFs using InPheRNo compared to the context-restricted network analysis. For each cancer type, 100 TFs with the most number of identified target genes are selected and are compared with the set of driver TFs of that cancer that are drivers of at most n_s other cancers. Color green shows the total number of such driver TFs for each cancer type in the DriverDBv2 database, color blue corresponds to number of cancer-specific driver TFs identified by InPheRNo and red represents driver TFs identified using context-restricted network analysis. The p-values are calculated using a hypergeometric test. (a) Results corresponding to $n_s = 2$. (b) Results corresponding to $n_s = 3$.

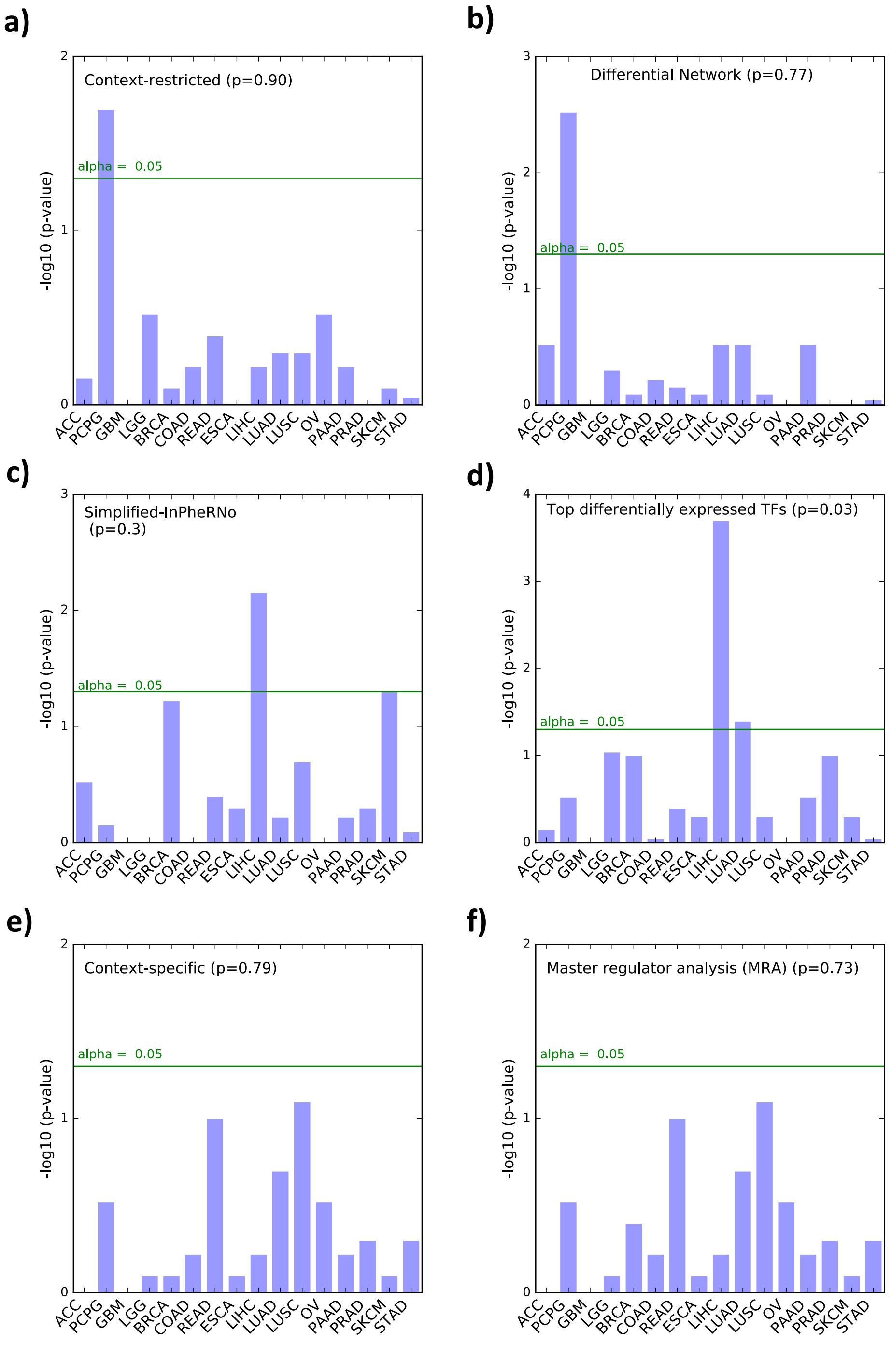

Supplementary Figure 7: Cancer-specificity of driver TFs identified using different methods. The bars represent the –log10 (p) of enrichment (hypergeometric test) of 100 TFs identified using different methods with driver TFs of that cancer type (annotated in the DriverDBv2 database) that are drivers of at most $n_s = 2$ other cancers. The p-value shown in parenthesis represents the Meta p-value calculated using Fisher's method. (a) Results correspond to 100 TFs with most identified targets reconstructed using context-restricted network analysis. (b) Results correspond to 100 TFs with most identified targets reconstructed using differential network analysis. (c) Results correspond to 100 TFs with most identified targets reconstructed by simplified-InPheRNo. (d) Results correspond to 100 TFs that were most differentially expressed in that cancer type compared to other cancers. (e) Results correspond to 100 TFs with most identified targets reconstructed using context-specific network analysis. (f) Results correspond to 100 key TFs identified using master regulator analysis (MRA).

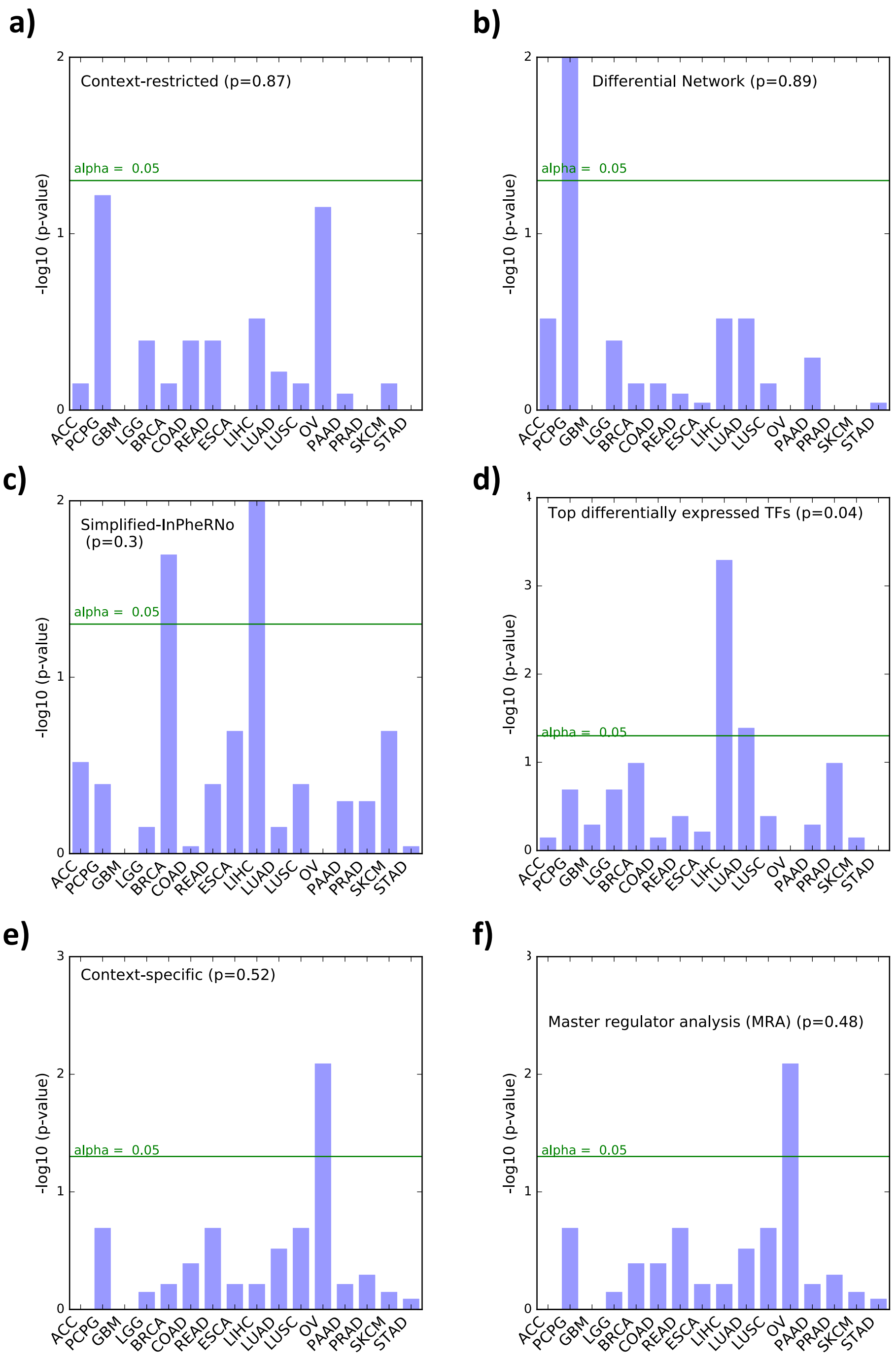

Supplementary Figure 8: Cancer-specificity of driver TFs identified using different methods. The bars represent the –log10 (p) of enrichment (hypergeometric test) of 100 TFs identified using different methods with driver TFs of that cancer type (annotated in the DriverDBv2 database) that are drivers of at most $n_s = 3$ other cancers. The p-value shown in parenthesis represents the Meta p-value calculated using Fisher's method. (a) Results correspond to 100 TFs with most identified targets reconstructed using context-restricted network analysis. (b) Results correspond to 100 TFs with most identified targets reconstructed using differential network analysis. (c) Results correspond to 100 TFs with most identified targets reconstructed by simplified-InPheRNo. (d) Results correspond to 100 TFs that were most differentially expressed in that cancer type compared to other cancers. (e) Results correspond to 100 TFs with most identified targets reconstructed using context-specific network analysis. (f) Results correspond to 100 key TFs identified using master regulator analysis (MRA).

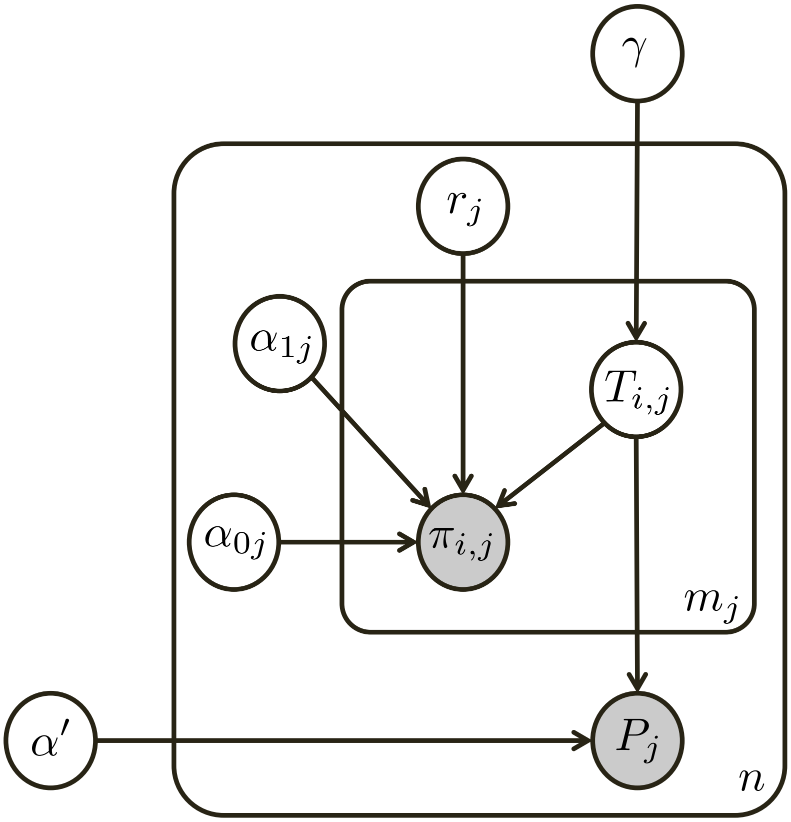

Supplementary Figure 9: The probabilistic graphical model used in InPheRNo shown using the plate notation. Color grey is used to denote the observed variables. In this model, $T_{i,j}$ is a binary random variable and $T_{i,j} = 1$ implies that TF i regulates gene j so as to affect the phenotype, and $T_{i,j} = 0$ indicates its logical complement. Note that n represents the total number of genes and m_j denotes the number of candidate TFs identified previously using an Elastic Net model. For a complete description of the parameters and their distributions see Methods.

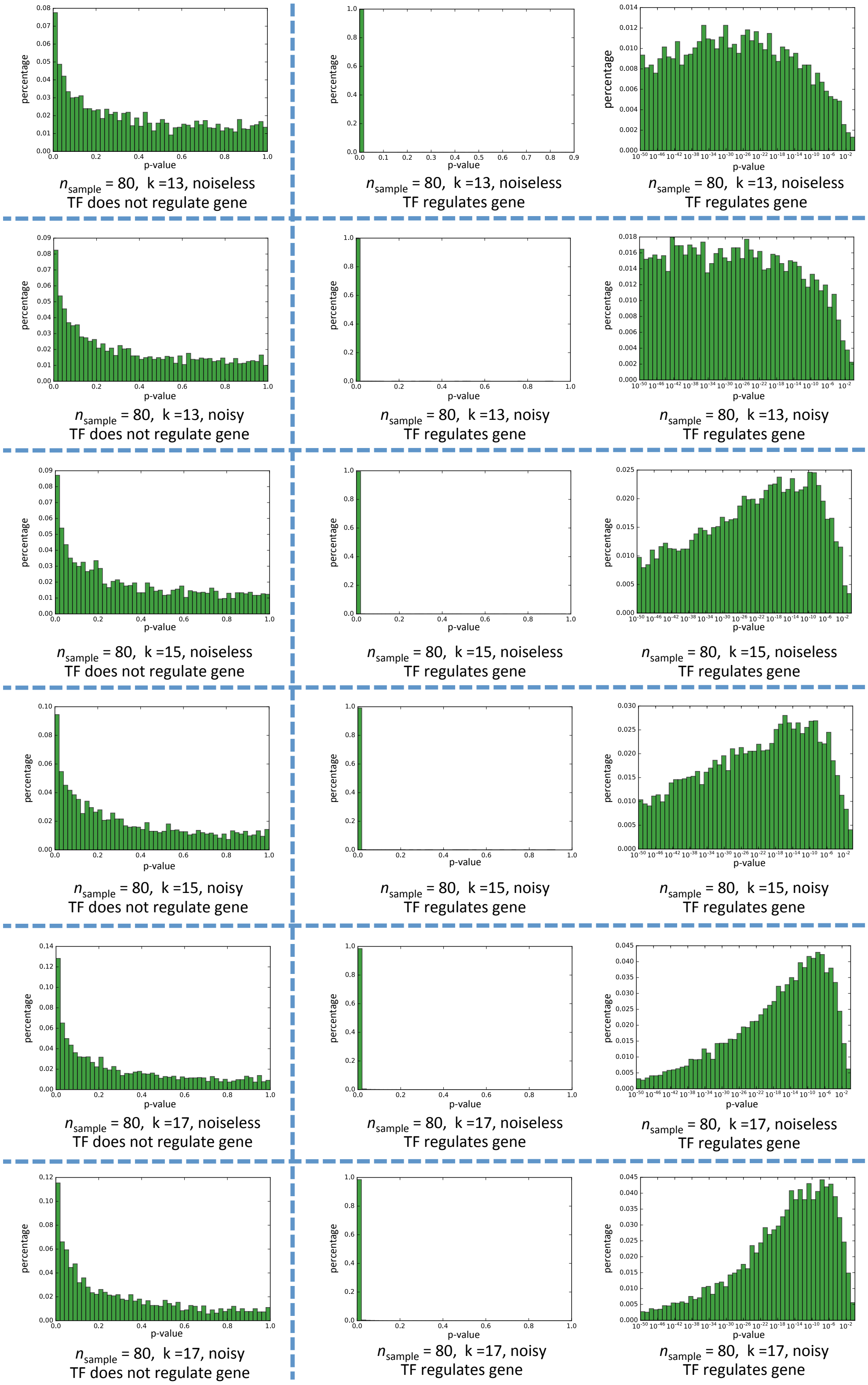

Supplementary Figure 10: Histogram of pseudo p-values of gene-TF associations obtained by applying the two-stage procedure used in InPheRNo to simulated data for $n_{\text{sample}} = 80$. Left column corresponds to the case in which a TF does not regulate a gene and the right column corresponds to the case in which a TF regulates a gene. A semilog plot is used in the most right column to show values between 1E-50 and 1. The rows correspond to different choices of k and noise.

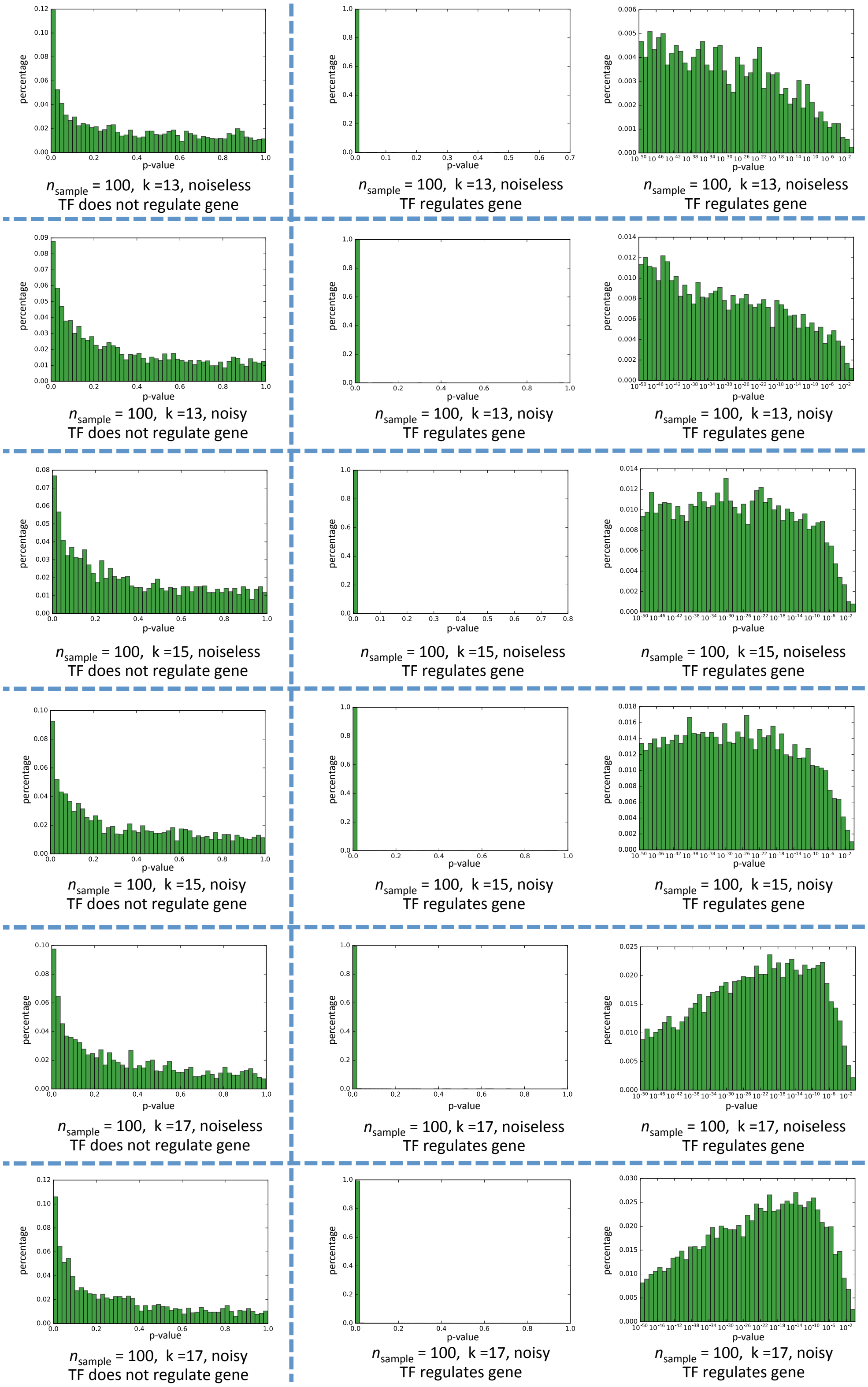

Supplementary Figure 11: Histogram of pseudo p-values of gene-TF associations obtained by applying the two-stage procedure used in InPheRNo to simulated data for $n_{\text{sample}} = 100$. Left column corresponds to the case in which a TF does not regulate a gene and the right column corresponds to the case in which a TF regulates a gene. A semilog plot is used in the most right column to show values between 1E-50 and 1. The rows correspond to different choices of k and noise.

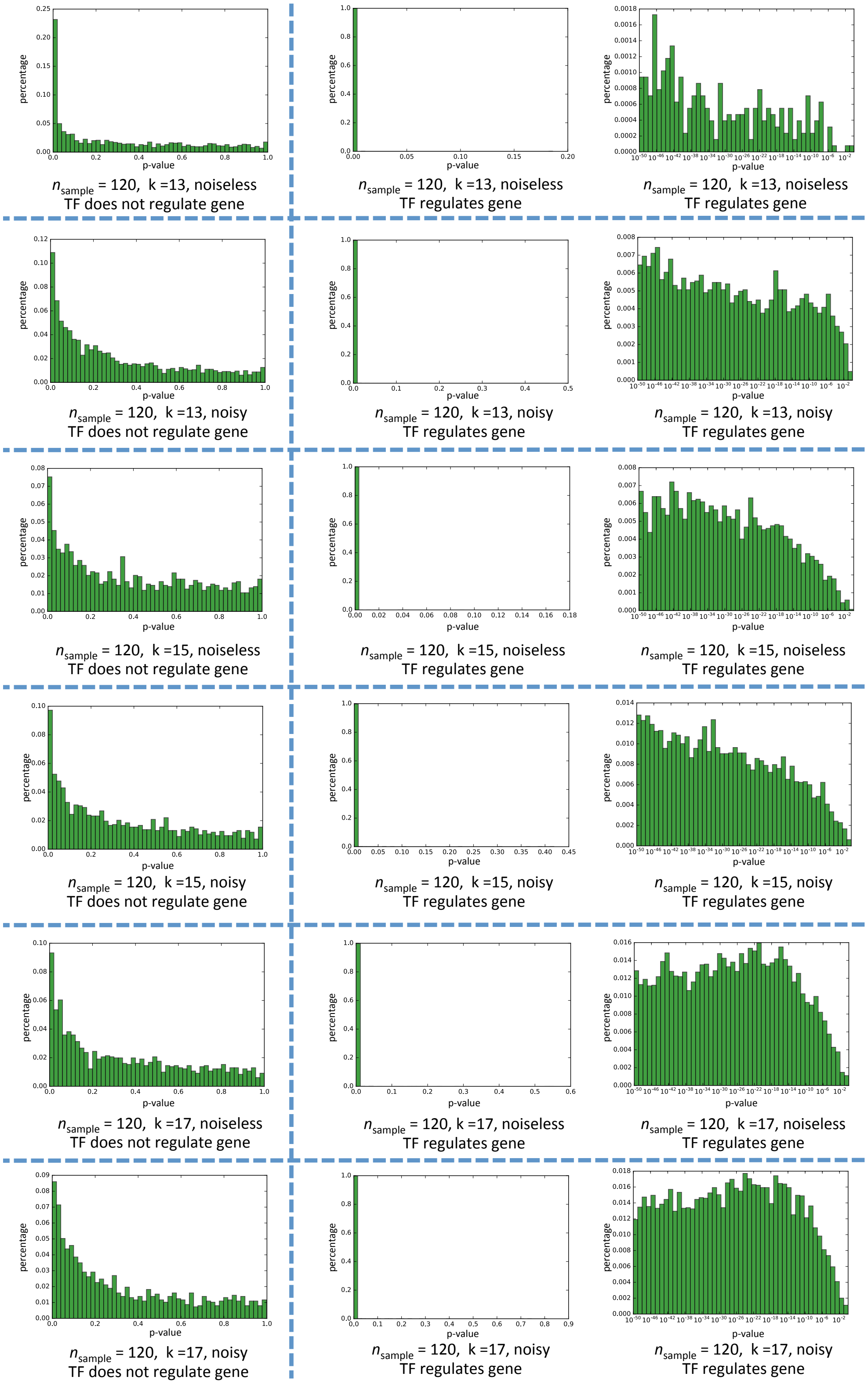

Supplementary Figure 12: Histogram of pseudo p-values of gene-TF associations obtained by applying the two-stage procedure used in InPheRNo to simulated data for $n_{\text{sample}} = 120$. Left column corresponds to the case in which a TF does not regulate a gene and the right column corresponds to the case in which a TF regulates a gene. A semilog plot is used in the most right column to show values between 1E-50 and 1. The rows correspond to different choices of k and noise.

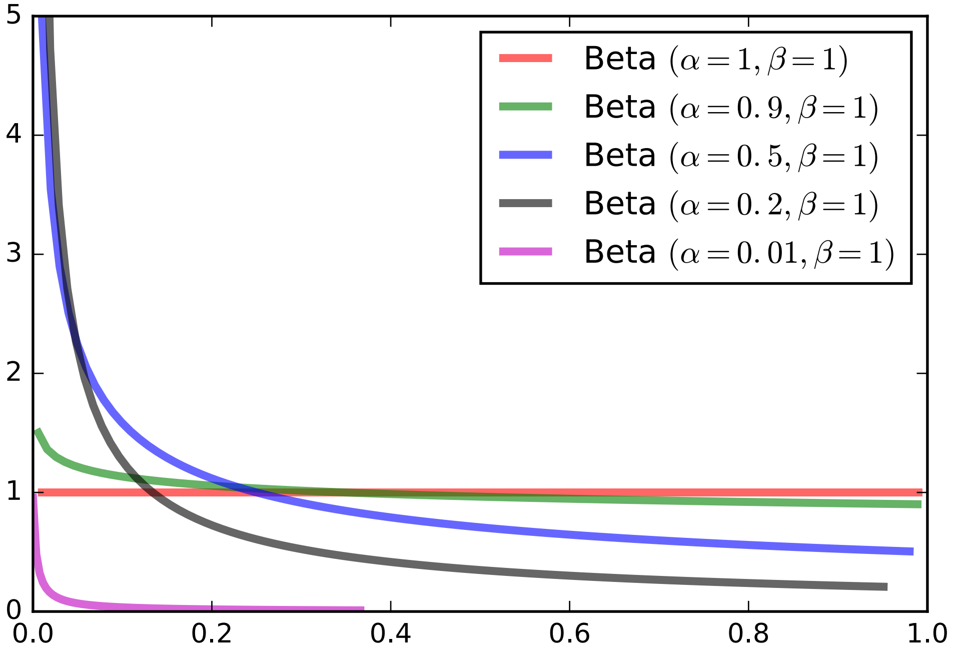

Supplementary Figure 13: Probability distribution function (PDF) of several Beta(α, β=1) distributions for different values of α. A smaller value of α results in a distribution that is more biased towards small values.

References:

1 Pedregosa, F. *et al.* Scikit-learn: Machine learning in Python. *Journal of machine learning research* **12**, 2825-2830, (2011).

2 Geier, F., Timmer, J. & Fleck, C. Reconstructing gene-regulatory networks from time series, knock-out data, and prior knowledge. *BMC Syst Biol* **1**, 11, (2007).

3 Chandrasekaran, S. *et al.* Behavior-specific changes in transcriptional modules lead to distinct and predictable neurogenomic states. *Proc Natl Acad Sci U S A* **108**, 18020-18025, (2011).

4 Emad, A. & Milenkovic, O. CaSPIAN: a causal compressive sensing algorithm for discovering directed interactions in gene networks. *PLoS One* **9**, e90781, (2014).

5 Patil, A., Huard, D. & Fonnesbeck, C. J. PyMC: Bayesian Stochastic Modelling in Python. *J Stat Softw* **35**, 1-81, (2010).

6 Goldman, M. *et al.* The UCSC Xena platform for public and private cancer genomics data visualization and interpretation. *bioRxiv*, 326470, (2019).

7 Grossman, R. L. *et al.* Toward a Shared Vision for Cancer Genomic Data. *N Engl J Med* **375**, 1109-1112, (2016).

8 McCarthy, D. J., Chen, Y. & Smyth, G. K. Differential expression analysis of multifactor RNA-Seq experiments with respect to biological variation. *Nucleic Acids Res* **40**, 4288-4297, (2012).

9 Law, C. W., Chen, Y., Shi, W. & Smyth, G. K. voom: Precision weights unlock linear model analysis tools for RNA-seq read counts. *Genome Biol* **15**, R29, (2014).

10 Rubio-Perez, C. *et al.* In silico prescription of anticancer drugs to cohorts of 28 tumor types reveals targeting opportunities. *Cancer Cell* **27**, 382-396, (2015).

11 Chung, I. F. *et al.* DriverDBv2: a database for human cancer driver gene research. *Nucleic Acids Res* **44**, D975-979, (2016).

12 Ray, P. S. *et al.* FOXC1 is a potential prognostic biomarker with functional significance in basal-like breast cancer. *Cancer Res* **70**, 3870-3876, (2010).
13 Wang, J. *et al.* FOXC1 regulates the functions of human basal-like breast cancer cells by activating NF-kappaB signaling. *Oncogene* **31**, 4798-4802, (2012).
14 Jensen, T. W. *et al.* Diagnosis of Basal-Like Breast Cancer Using a FOXC1-Based Assay. *J Natl Cancer Inst* **107**, (2015).
15 Elian, F. A., Yan, E. & Walter, M. A. FOXC1, the new player in the cancer sandbox. *Oncotarget* **9**, 8165-8178, (2018).
16 Nakshatri, H. & Badve, S. FOXA1 in breast cancer. *Expert Rev Mol Med* **11**, e8, (2009).
17 Habashy, H. O. *et al.* Forkhead-box A1 (FOXA1) expression in breast cancer and its prognostic significance. *Eur J Cancer* **44**, 1541-1551, (2008).
18 Badve, S. *et al.* FOXA1 expression in breast cancer--correlation with luminal subtype A and survival. *Clin Cancer Res* **13**, 4415-4421, (2007).
19 Emad, A. *et al.* An epithelial-mesenchymal-amoeboid transition gene signature reveals molecular subtypes of breast cancer progression and metastasis. *bioRxiv 219410*, (2017).
20 Mani, S. A. *et al.* Mesenchyme Forkhead 1 (FOXC2) plays a key role in metastasis and is associated with aggressive basal-like breast cancers. *Proc Natl Acad Sci U S A* **104**, 10069-10074, (2007).
21 Cai, J. *et al.* FOXF2 suppresses the FOXC2-mediated epithelial-mesenchymal transition and multidrug resistance of basal-like breast cancer. *Cancer Lett* **367**, 129-137, (2015).
22 Lu, X. F. *et al.* FoxM1 is a promising candidate target in the treatment of breast cancer. *Oncotarget* **9**, 842-852, (2018).
23 Tan, Y. *et al.* Identification of FOXM1 as a specific marker for triplenegative breast cancer. *Int J Oncol* **54**, 87-97, (2019).
24 Grimm, D. *et al.* The role of SOX family members in solid tumours and metastasis. *Semin Cancer Biol*, (2019).
25 Tang, H. *et al.* SOX8 acts as a prognostic factor and mediator to regulate the progression of triple-negative breast cancer. *Carcinogenesis*, (2019).
26 Kotarba, G., Krzywinska, E., Grabowska, A. I., Taracha, A. & Wilanowski, T. TFCP2/TFCP2L1/UBP1 transcription factors in cancer. *Cancer Lett* **420**, 72-79, (2018).
27 Ali, S. & Coombes, R. C. Estrogen receptor alpha in human breast cancer: occurrence and significance. *J Mammary Gland Biol Neoplasia* **5**, 271-281, (2000).
28 Holst, F. *et al.* Estrogen receptor alpha (ESR1) gene amplification is frequent in breast cancer. *Nat Genet* **39**, 655-660, (2007).
29 Robinson, D. R. *et al.* Activating ESR1 mutations in hormone-resistant metastatic breast cancer. *Nat Genet* **45**, 1446-1451, (2013).
30 Bastien, R. R. *et al.* PAM50 breast cancer subtyping by RT-qPCR and concordance with standard clinical molecular markers. *BMC Med Genomics* **5**, 44, (2012).
31 Haibe-Kains, B. *et al.* A three-gene model to robustly identify breast cancer molecular subtypes. *J Natl Cancer Inst* **104**, 311-325, (2012).
32 Inoue, K. & Fry, E. A. Novel Molecular Markers for Breast Cancer. *Biomark Cancer* **8**, 25-42, (2016).
33 Chen, J. & Chen, X. MYBL2 Is Targeted by miR-143-3p and Regulates Breast Cancer Cell Proliferation and Apoptosis. *Oncol Res* **26**, 913-922, (2018).
34 Thorner, A. R. *et al.* In vitro and in vivo analysis of B-Myb in basal-like breast cancer. *Oncogene* **28**, 742-751, (2009).
35 Li, Y. *et al.* c-Myb Enhances Breast Cancer Invasion and Metastasis through the Wnt/beta-Catenin/Axin2 Pathway. *Cancer Res* **76**, 3364-3375, (2016).
36 Knopfova, L. *et al.* Transcription factor c-Myb inhibits breast cancer lung metastasis by suppression of tumor cell seeding. *Oncogene* **37**, 1020-1030, (2018).

37 Liu, X., Xu, Y., Han, L. & Yi, Y. Reassessing the Potential of Myb-targeted Anti-cancer Therapy. *J Cancer* **9**, 1259-1266, (2018).

38 Vimala, K., Sundarraj, S., Sujitha, M. V. & Kannan, S. Curtailing overexpression of E2F3 in breast cancer using siRNA (E2F3)-based gene silencing. *Arch Med Res* **43**, 415-422, (2012).

39 Lee, M., Oprea-Ilies, G. & Saavedra, H. I. Silencing of E2F3 suppresses tumor growth of Her2+ breast cancer cells by restricting mitosis. *Oncotarget* **6**, 37316-37334, (2015).

40 Mehra, R. *et al.* Identification of GATA3 as a breast cancer prognostic marker by global gene expression meta-analysis. *Cancer Res* **65**, 11259-11264, (2005).

41 Dydensborg, A. B. *et al.* GATA3 inhibits breast cancer growth and pulmonary breast cancer metastasis. *Oncogene* **28**, 2634-2642, (2009).

42 Yan, W., Cao, Q. J., Arenas, R. B., Bentley, B. & Shao, R. GATA3 inhibits breast cancer metastasis through the reversal of epithelial-mesenchymal transition. *J Biol Chem* **285**, 14042-14051, (2010).

43 Zhu, L., Pan, R., Zhou, D., Ye, G. & Tan, W. BCL11A enhances stemness and promotes progression by activating Wnt/beta-catenin signaling in breast cancer. *Cancer Manag Res* **11**, 2997-3007, (2019).

44 Chen, F., Luo, N., Hu, Y., Li, X. & Zhang, K. MiR-137 Suppresses Triple-Negative Breast Cancer Stemness and Tumorigenesis by Perturbing BCL11A-DNMT1 Interaction. *Cell Physiol Biochem* **47**, 2147-2158, (2018).

45 Khaled, W. T. *et al.* BCL11A is a triple-negative breast cancer gene with critical functions in stem and progenitor cells. *Nat Commun* **6**, 5987, (2015).

46 Kokoska, S. & Zwillinger, D. *CRC standard probability and statistics tables and formulae*. (Crc Press, 1999).

47 Wasserman, L. & Roeder, K. High Dimensional Variable Selection. *Ann Stat* **37**, 2178-2201, (2009).

48 Meinshausen, N. & Bühlmann, P. Stability selection. *Journal of the Royal Statistical Society: Series B (Statistical Methodology)* **72**, 417-473, (2010).

49 Lockhart, R., Taylor, J., Tibshirani, R. J. & Tibshirani, R. A Significance Test for the Lasso. *Ann Stat* **42**, 413-468, (2014).

50 Margolin, A. A. *et al.* ARACNE: an algorithm for the reconstruction of gene regulatory networks in a mammalian cellular context. *BMC Bioinformatics* **7 Suppl 1**, S7, (2006).
